# Feeling Socially Connected and Focusing on Growth: Relationships With Wellbeing During a Major Holiday in the COVID-19 Pandemic

**DOI:** 10.3389/fpsyg.2021.710491

**Published:** 2021-08-26

**Authors:** Leigh Ann Vaughn, Patricia G. Burkins, Rachael D. Chalachan, Janak K. Judd, Chase A. Garvey, John W. Luginsland

**Affiliations:** ^1^Psychology Department, Ithaca College, Ithaca, NY, United States; ^2^Confluent Sciences, LLC, Albuquerque, NM, United States

**Keywords:** satisfaction, positive affect, negative affect, regulatory fit, ideals, duties, caution, self-control

## Abstract

Numerous major holidays celebrate socially gathering in person. However, in major holidays that happened during the pandemic, desires to nurture relationships and maintain holiday traditions often conflicted with physical distancing and other measures to protect against COVID-19. The current research sought to understand wellbeing during American Thanksgiving in 2020, which happened 8months into the COVID-19 pandemic, after months of physical distancing and stay-at-home orders. American Thanksgiving is a major holiday not limited to any religion. We asked 404 American adults how they spent Thanksgiving Day and to report on their experiences of that day. Predictors of wellbeing that we drew from self-determination theory were satisfaction of the fundamental needs for social connection (relatedness), for doing what one really wants (autonomy), and feeling effective (competence). The predictors of wellbeing that we drew from regulatory focus theory were a focus on growth (promotion), and a focus on security (prevention). We found that feeling socially connected and focusing on growth related most strongly to wellbeing. Additionally, participants who saw even one other person face-to-face reported significantly higher relatedness satisfaction, promotion focus, and wellbeing than those who did not. Our research could help construct persuasive messages that encourage nurturing close relationships at major holidays while remaining safe against the virus.

## Introduction

Measures to slow the spread of the COVID-19 virus during the pandemic included quarantines and stay-at-home orders, and even when those orders were not in place, many government agencies and other institutions encouraged physical distancing and staying at home. Months of uncertainty about how and when the pandemic would end, as well as economic and social costs of slowing the spread of the virus, strained people’s sense of social belonging and their wellbeing (e.g., Dawel et al., 2020; Gray et al., 2020; Vanderweele et al., 2020; Okabe-Miyamoto et al., 2021). In the United States, for example, negative mental health consequences of quarantining, social distancing, and closures were widespread by fall of 2020 (e.g., Mental Health America, 2020; National Center for Health Statistics, 2021; Panchal et al., 2021). The quality and quantity of social relationships were key factors for wellbeing during the pandemic (Okabe-Miyamoto et al., 2021), but measures to slow the spread of the virus presented threats to felt social connection and wellbeing (Sikali, 2020). Many major holidays celebrate socially connecting in person and could offer welcome respite from social isolation. However, for many people, the desire to nurture relationships and to secure important holiday traditions conflicted with the desire to protect the self and others from the virus. Wellbeing in these challenging circumstances is important to understand for resilience, both for the current pandemic and for future large-scale epidemics, which may become more common (Hilsenrath, 2020). The current study is basic research that examined predictors of wellbeing in the American holiday of Thanksgiving in 2020. To do so, it extended research on wellbeing in major holidays as well as two major theories pertaining to wellbeing, self-determination theory (Deci and Ryan, 2000; Ryan and Deci, 2017) and regulatory focus theory (Higgins, 1997; Scholer et al., 2019a,b).

### Holidays and Wellbeing

Major holidays are important for maintaining and growing close connections with family members, and people often experience increased wellbeing at these times (e.g., Fiese et al., 2002; Kasser and Sheldon, 2002; Páez et al., 2011; Allan et al., 2013; Hanke et al., 2016). It is common for family members to travel long distances to gather and enjoy traditions that emphasize the importance of family and the occasion. At Thanksgiving in the United States, these traditions often include gathering with extended family, expressing gratitude for relationships and other good things in life, having a holiday dinner, watching a parade on TV, playing games, and watching football after dinner (Thanksgiving (United States), 2020). Gallup polls consistently have shown that for Americans, Thanksgiving is one of the happiest days of the year with the least stress (Witters, 2011; McCarthy, 2015). It is also a major holiday that is not limited to any religion (unlike Christmas, for example, cf. Kasser and Sheldon, 2002).

American Thanksgiving occurs on the fourth Thursday of November (Thanksgiving (United States), 2020), which was several months into the COVID-19 pandemic in 2020. In fall 2020, the Centers for Disease Control and Prevention and others recommended revising traditional Thanksgiving celebrations to be safer, such as by keeping gatherings small, self-quarantining, and avoiding travel (Grantham-Philips, 2020; Sullivan, 2020; Centers for Disease Control and Prevention, 2021). Many Americans limited their holiday celebrations (Reuters, 2020; Whitcomb and Layne, 2020), and 57–61% of public opinion poll respondents said they changed their Thanksgiving plans due to COVID-19 (Abidi and Gramlich, 2020; Thomas, 2020). Still, amidst a surge in cases, more Americans traveled for Thanksgiving than at any time since the beginning of the pandemic (Caspiani and Borter, 2020; Tanne, 2020; Trotta and Layne, 2020). Many felt trepidation but made the trip anyway, because they had not seen their family members in a long time (Caspiani and Borter, 2020; Trotta and Layne, 2020). There are countless things about American Thanksgiving that could make people feel satisfied and happy, but for many people, Thanksgiving during the COVID-19 pandemic in 2020 was relatively subdued (Reuters, 2020; Whitcomb and Layne, 2020). Many Americans felt conflicted about how to celebrate the holiday. For example, how could they feel close to family members, maintain family traditions, and have fun, while also remaining vigilant against COVID-19?

In contrast to earlier longitudinal research that has examined whether holidays make people happy (e.g., Gilbert and Abdullah, 2004; Nawijn et al., 2010; Allan et al., 2013), the current cross-sectional research sought to understand what experiences could relate most strongly to wellbeing during Thanksgiving in the COVID-19 pandemic. We applied self-determination theory (Deci and Ryan, 2000; Ryan and Deci, 2017) because it proposes that relatedness (feeling close and connected to others) is one of the three fundamental psychological needs that are crucial for wellbeing. Relatedness is also a key experience that people seek on Thanksgiving (e.g., Fiese et al., 2002; Kasser and Sheldon, 2002; Páez et al., 2011; Allan et al., 2013; Hanke et al., 2016). We also assessed satisfaction of the other two needs that self-determination theory proposes are fundamental to wellbeing: autonomy and competence. This is because the needs for autonomy, competence, and relatedness mutually satisfy each other (e.g., Ryan and Deci, 2017). The other theory we applied was regulatory focus theory (Higgins, 1997, 1998; Scholer et al., 2019a) because it distinguishes between two self-regulatory orientations that relate to wellbeing (Lanaj et al., 2012; Koopmann et al., 2016; Zhang et al., 2019; Wu and Chen, 2020) and that are important in responses to COVID-19 (Vaughn et al., 2020). These orientations are promotion and prevention focus.

Given the value normally placed on gathering physically with others on Thanksgiving and other major holidays (e.g., Fiese et al., 2002; Kasser and Sheldon, 2002; Páez et al., 2011; Allan et al., 2013; Hanke et al., 2016), we expected that people who spent Thanksgiving alone would report lower wellbeing and less satisfying connections with others. In addition to testing these hypotheses, we explored how seeing no one else in person *versus* one, a few, or more people in person related to satisfaction of other basic needs and to strength of promotion and prevention focus on Thanksgiving.

### Self-Determination Theory’s Basic Needs and Wellbeing on Thanksgiving

Self-determination theory proposes that all people have psychological needs for autonomy, relatedness, and competence (e.g., Deci and Ryan, 2000; Ryan and Deci, 2017). It proposes that satisfaction of all three needs is essential for wellbeing and optimal psychological functioning and that when any of these needs are frustrated, the individual suffers. The need for autonomy is do what one really wants, the need for competence is to feel effective at challenging tasks, and the need for relatedness to feel close and connected to others (also see Baumeister and Leary, 1995). Self-determination theory also proposes that the three needs are interdependent: In general, satisfaction of one need facilitates satisfaction of the other two (e.g., Ryan and Deci, 2017). For example, if people do not feel able to be their true selves or competent within romantic relationships, they likely will not feel very emotionally close to their partners (Knee et al., 2013). Research shows that greater satisfaction of all three needs correlates very strongly with wellbeing, feelings of personal integrity, and feeling volitional (for reviews, see Deci and Ryan, 2000; Ryan and Deci, 2017). Satisfactions of these needs tend to be highly correlated (e.g., Sheldon and Hilpert, 2012; Chen et al., 2015; Vaughn, 2017, 2019; Vaughn et al., 2020), and balanced satisfaction of these needs appears to be important for wellbeing, both within persons (Sheldon and Niemiec, 2006) and between life domains (Milyavskaya et al., 2009). With that said, “in different settings, any one of the three needs will emerge to ‘take the lead’ in terms of its association with wellness outcomes, even as the other two remain important” (Ryan and Deci, 2017, p. 247).

Relatedness support is highly valued at major holidays (e.g., Fiese et al., 2002; Kasser and Sheldon, 2002; Páez et al., 2011; Hanke et al., 2016). Thus, we predicted that higher relatedness satisfaction would relate to wellbeing. Research on wellbeing at major holidays has not emphasized support for autonomy or competence, so our examination of relationships between these other two needs and wellbeing at Thanksgiving was exploratory.

### Regulatory Focus and Wellbeing on Thanksgiving

Regulatory focus theory distinguishes between two self-regulatory orientations: promotion focus and prevention focus (Higgins, 1997, 1998). These orientations are independent of each other (e.g., Higgins et al., 2001; Haws et al., 2010) and the strength of each orientation differs temporarily, depending on the immediate circumstances, as well as chronically between individuals (e.g., Higgins et al., 2001). According to regulatory focus theory, prevention focus serves the need for security, whereas promotion focus serves the need for growth (for reviews, see Higgins, 1997; 1998; Molden et al., 2007; Scholer et al., 2019a,b). Individuals in a promotion focus seek to approach successes and avoid failures, which they view as gains and nongains, and to be eager to fulfill hopes and aspirations. Those in a prevention focus also seek to approach successes and avoid failures, which they view as nonlosses and losses, and to be vigilant to protect the self and others and to fulfill duties and obligations.

We expected that on Thanksgiving, promotion focus would relate more strongly than prevention focus to positive wellbeing, which is a common finding (cf. Grant and Higgins, 2003 Lanaj et al., 2012; Koopmann et al., 2016; Zhang et al., 2019; Wu and Chen, 2020). In general, promotion focus feels better than prevention focus does (e.g., Vaughn, 2017, 2019; Scholer et al., 2019b; Vaughn et al., 2020). In part, this difference reflects how promotion-focused goals are oriented toward gains and away from their absence, whereas prevention-focused goals are oriented away from losses and toward their absence (Higgins, 1997;^1998^; Scholer et al., 2019a). This difference also reflects how the preferred strategies in promotion- and prevention-focused goal pursuit tend to feel. Eagerness, which fits promotion, involves thinking more positively than vigilance, which fits prevention (for reviews, see Higgins, 2000; Scholer et al., 2019b). Thus, compared with people in a prevention focus, those in a promotion focus experience more intense positive affect (Higgins, 1997, 1998; Idson et al., 2000), selectively attend more to positive information (Yoon et al., 2012), and recall more positive information about the self (Scholer et al., 2014). Additionally, studies that have used Linguistic Inquiry and Word Count software (LIWC; Pennebaker et al., 2015) to analyze thousands of descriptions of promotion- and prevention-focused experiences have found that participants describe promotion experiences more positively than prevention experiences (Vaughn, 2018, 2019; Vaughn et al., 2020; cf. Scholer et al., 2010). Research also has shown that participants report more satisfaction of needs for autonomy, competence, and relatedness in promotion-focused experiences than in prevention-focused ones (Vaughn, 2017, 2019; Kim et al., 2019).^1^ With that said, people tend not to view prevention-focused experiences as need thwarting (Vaughn, 2017, 2019; Kim et al., 2019; Vaughn et al., 2020), and LIWC analyses of prevention-focused experiences show that the emotional tone of prevention experiences is much more positive than of experiences that are explicitly low in need satisfaction (Vaughn, 2019).

### Relative Strength of Promotion and Prevention on Thanksgiving

In the COVID-19 pandemic, prevention was encouraged (Centers for Disease Control and Prevention, 2020b,c, 2021). Research in the first month of the COVID-19 pandemic showed that participants judged prevention focus to be more useful than promotion focus for responding to the virus (Vaughn et al., 2020).

In the current research, we examined whether participants reported being more prevention-focused than promotion-focused during Thanksgiving, 2020. We did not have strong predictions about the relative strength of promotion and prevention focus. On the one hand, prevention focus could continue to be stronger than promotion focus on this Thanksgiving because a surge in COVID-19 cases in the United States was happening at that time (Tanne, 2020), which could prompt many people to remain vigilant. Additionally, prevention focus could continue to be strong because maintaining holiday traditions sometimes can feel obligatory (e.g., Hanke et al., 2016). On the other hand, promotion focus could be stronger than prevention focus on this Thanksgiving because the holiday emphasizes nurturing relationships and having fun. This could be a welcome break from the stress and exhaustion of keeping vigilant and socially distanced over the previous months. Thus, we did not have a strong hypothesis about whether promotion or prevention focus would be stronger on this day.

### The Current Research

This study happened on the Friday after Thanksgiving Day, 2020, which is part of the Thanksgiving holiday (Thanksgiving (United States), 2020). We asked participants to describe what they did on Thanksgiving Day. Then, we asked them to report their need satisfaction, regulatory focus, and wellbeing on this Thanksgiving. Consistent with other research on wellbeing at holidays and in general (e.g., Kasser and Sheldon, 2002; Sheldon and Hilpert, 2012), our measures of overall wellbeing at Thanksgiving were positive affect, low negative affect, and satisfaction with Thanksgiving (cf. satisfaction with Christmas; Kasser and Sheldon, 2002).

We predicted that at Thanksgiving in 2020, participants who were higher in relatedness satisfaction and promotion focus would report higher wellbeing, whereas participants who were higher in prevention focus would report lower wellbeing. Additionally, we predicted that participants who spent Thanksgiving physically alone would report lower wellbeing and less relatedness support than those who did not. We did numerous exploratory analyses, as well. For example, we explored how many participants reported being satisfied with Thanksgiving and whether more participants reported higher prevention focus than promotion focus that day. We also explored how autonomy and competence satisfaction related to wellbeing, and whether participants who did *versus* not see anyone else face-to-face differed on autonomy, competence, promotion, or prevention focus.

## Materials and Methods

### Reporting

We obtained approval from the Ethics Committee of Ithaca College. The procedures used in this study adhere to the tenets of the Declaration of Helsinki. We report how we determined our sample size, as well as all data exclusions, all manipulations, and all measures in the study. This study was not preregistered. We used SPSS 26 and JASP (JASP Team, 2020, Version 0.13.1). The data files, data dictionaries, and verbatim materials for the current investigation are available at https://osf.io/fy6rc/. We conducted sensitivity power analyses with G*Power (Faul et al., 2007), and the results of these power analyses are in the relevant parts of the results section.

### Participants and Recruiting

Data collection happened on November 27, 2020, which was the day after American Thanksgiving. The target sample size was 400 participants, based on available research funds. We recruited participants through Prolific (https://www.prolific.co/), a participant pool for online studies that has been found to provide high-quality data for social science research (Peer et al., 2017; Palan and Schitter, 2018). In it, we set the following criteria for participation. Participants had to be at least 18years old, live in the United States, and have English as their first language. They also had to have an acceptance rate on Prolific studies of at least 95% and to have not done any of our lab’s prior studies on Prolific. To reduce variability in written responses, they had to do the study on a tablet or desktop computer rather than a phone. The study took approximately 8min, so respondents received USD $1.27 for participating.

We excluded 48 cases either because they reported not living in the United States (*n*=43) or because the latitude/longitude data automatically collected by Qualtrics indicated a location outside the United States (*n*=5). Most of these exclusions happened because initially, we accidentally opened the study to Canadians as well as Americans. Canadian Thanksgiving was on October 12, 2020, and it is a relatively minor holiday (The Old Farmer’s Almanac, 2020). Most of the excluded participants lived in Canada. These excluded cases are in a separate data file at https://osf.io/fy6rc/. We exceeded the target sample size because we anticipated needing to exclude more cases.

In the final sample of 404 participants, 234 (57.9%) identified as female.^2^ Mean age was 32.9years (*SD*=12.76). Participants selected the racial and ethnic categories to which they belonged; 320 selected White (79.2%), 50 selected Asian (12.4%), 37 selected Black or African American (9.2%), 29 selected Hispanic or Latinx (7.2%), five selected Native American or Alaska Native (1.2%), and eight selected “other” (2.0%). The methodology and data files at https://osf.io/fy6rc/ contain the other background information we collected, including education and occupation.

## Materials

### Writing Task

The first page of stimulus materials was titled “How You Spent this Thanksgiving Day.” It stated, “First, we would like to learn about how you spent this Thanksgiving Day, 2020. This is a general question, and you can write about your thoughts, feelings, and/or behaviors. Please take a minute or two and write about how you spent this Thanksgiving Day.”

### Need Satisfaction on Thanksgiving

The second page of stimulus materials automatically piped in what the participants wrote on the first page and asked them to rate how much they agreed with statements about how they spent this Thanksgiving Day (1=*strongly disagree* and 7=*strongly agree*). This page contained the Balanced Measure of Psychological Needs (Sheldon and Hilpert, 2012), which contains three six-item subscales that measure satisfaction of autonomy (e.g., “I was really doing what interested me” and “There were people telling me what I had to do”; reverse-scored), competence (e.g., “I took on and mastered hard challenges” and “I did stupid things that made me feel incompetent”; reverse-scored), and relatedness (e.g., “I felt close and connected with other people who were important to me” and “I feel unappreciated by one or more important people”; reverse-scored). After appropriate reverse scoring, we calculated an index for each subscale by taking the mean of the relevant items. Table 1 shows the Cronbach’s alphas for the final indexes of regulatory focus, need satisfaction, affect, and satisfaction with Thanksgiving.^3^

**Table 1:** Correlations, Cronbach’s alphas, means, and standard deviations.

S. No.	Variable	1	2	3	4	5	6	7	8	9
1.	Promotion	–								
2.	Caution	0.03	–							
3.	Duties	0.32^**^	0.27^**^	–						
4.	Autonomy	0.58^**^	−0.04	0.01	–					
5.	Competence	0.54^**^	0.15^**^	0.39^**^	0.49^**^	–				
6.	Relatedness	0.69^**^	0.00	0.29^**^	0.60^**^	0.55^**^	–			
7.	Pos. affect	0.82^**^	0.01	0.30^**^	0.60^**^	0.52^**^	0.78^**^	–		
8.	Neg. affect	−0.59^**^	0.10^*^	−0.15^**^	−0.58^**^	−0.48^**^	−0.68^**^	−0.77^**^	–	
9.	SWTS	0.77^**^	−0.07	0.24^**^	0.56^**^	0.44^**^	0.70^**^	0.80^**^	−0.65^**^	–
Cronbach’s *α*		0.87	0.72	0.69	0.81	0.65	0.82	0.96	0.92	0.92
*M*		4.37	5.07	5.22	5.18	4.98	5.42	5.21	2.29	4.59
*SD*		(1.32)	(1.38)	(1.28)	(1.21)	(0.97)	(1.26)	(1.47)	(1.29)	(1.66)

*SWTS, Satisfaction with Thanksgiving Scale. M and SD represent mean and standard deviation, respectively*.

* *p<0.05*.

*and*

** *p<0.01*.

### Promotion and Prevention Focus on Thanksgiving

There was no existing measure of recalled regulatory focus from the previous day that assessed aspects of the promotion and prevention systems other than hopes/ideals for promotion and duties/oughts for prevention (cf. Vaughn, 2017, Study 2). Therefore, we developed a new scale for the current research by modifying the promotion and prevention measure in Vaughn et al.’s (2020) research. Because hopes/ideals and duties/oughts are the most common ways to operationally define promotion and prevention focus (e.g., Summerville and Roese, 2008; Hodis, 2017), we included items about these goals. We also included items based on research about regulatory focus and openness to new experiences (Vaughn et al., 2008), questionnaire measures of chronic and situational regulatory focus (Higgins et al., 2001; Lockwood et al., 2002; Ouschan et al., 2007; Wallace et al., 2009; Haws et al., 2010; Fay et al., 2019), and research on how regulatory focus relates to episodes of exploration and self-control (Manczak et al., 2014; Vaughn et al., 2020) as well as optimism and defensive pessimism (e.g., Grant and Higgins, 2003; Scholer et al., 2019a). Participants used this scale on the day after Thanksgiving to describe how they spent the previous day.

The third page of stimulus materials automatically piped in what the participants wrote on the first page. It asked participants to “Please indicate how much each of the following describes how you spent this Thanksgiving Day.” Participants responded on a 7-point scale (1=not at all and 7=very much). Eight items represented promotion (e.g., “Being spontaneous”) and eight represented prevention (e.g., “Exerting self-control”). Table 2 shows these items.

**Table 2:** Communalities and factor loadings from the exploratory factor analysis of regulatory focus items.

Item No.		Factor			Communalities	
		1	2	3	Initial	Extracted
14. I was excited.		**0.861**	0.027	0.052	0.723	0.777
4. I was enthusiastic.		**0.860**	0.069	0.035	0.729	0.785
6. I was optimistic.		**0.835**	0.021	0.047	0.676	0.728
12. I did what I ideally liked to.		**0.735**	−0.044	−0.041	0.547	0.517
8. I was spontaneous.		**0.614**	−0.050	−0.067	0.400	0.354
10. I avoided missing out on anything good.		**0.472**	0.021	0.233	0.360	0.341
11. I was careful.		−0.007	**0.810**	−0.036	0.466	0.634
13. I was cautious.		−0.224	**0.792**	−0.022	0.451	0.604
15. I exerted willpower.		0.209	**0.437**	0.001	0.400	0.268
3. I exerted self-control.		0.047	**0.416**	0.079	0.354	0.214
9. I did what was expected of me.		−0.143	−0.016	**1.033**	0.365	0.999
7. I fulfilled duties and obligations.		0.264	0.120	**0.462**	0.423	0.414

*N=401. Maximum likelihood exploratory factor analysis and direct oblimin rotation with delta=0. Loadings are from the pattern matrix, and loadings over 0.40 are in bold font. Factor 1 represents promotion, Factor 2 represents caution/self-control, and Factor 3 represents duties*.

Because this is the first study to use this new scale, we submitted the 16 regulatory focus items to an exploratory factor analysis using maximum likelihood estimation and direct oblimin rotation, with delta=0. The Kaiser-Meyer-Olkin measure showed that the sampling was adequate, KMO=0.831. Bartlett’s test of sphericity showed that the correlation structure was adequate for analyses, *χ*^2^ (120)=2552.19, *p*<0.001. These factors together accounted for 50.81% of the variance. Each item loaded >0.40 on only one factor, except for the following three items, which loaded <0.40 on each factor: “I avoided making mistakes,” “I thought through anything that could go wrong,” and “I avoided thinking about what could go wrong.” These questions were in positions 1, 5, and 16, respectively.

When we re-ran the factor analysis without these three items, KMO=0.822 and Bartlett’s test *χ*^2^ (78)=2552.19, *p*<0.001. Table 2 shows the pattern-matrix factor loadings and the communalities for the items. The resulting promotion and positive affect factors accounted for 52.93% of the variance. As expected, the promotion items loaded into one factor. However, the prevention items loaded into two factors: one for duties and the other for caution/self-control. The promotion factor correlated at *r*=0.26 with duties and at *r*=0.18 with caution/self-control. The duties and caution/self-control factors correlated with each other at *r*=0.36.^4^

### Positive and Negative Affect on Thanksgiving

The fourth page of stimulus materials automatically piped in what the participants wrote on the first page. It asked participants to “Please think about what you did and experienced on Thanksgiving Day. Then report how much you experienced each of the following feelings.” Participants responded on a 7-point scale (1=very slightly and 7=extremely). The six positive feelings (happy, positive, good, pleasant, joyful, and contented) and six negative feelings (sad, negative, bad, unpleasant, afraid, and angry) were the Scale of Positive and Negative Experience (Diener et al., 2010).^5^

### Satisfaction With Thanksgiving

The fifth page of stimulus materials automatically piped in what the participants wrote on the first page. It asked them, “How satisfied are you with how this Thanksgiving Day went?” For this measure, we adapted the five-item Satisfaction with Life Scale (Pavot and Diener, 1993) and asked participants to report on a 1 (strongly disagree) to 7 (strongly agree) scale their agreement with items, such as “I was satisfied with this Thanksgiving Day” (cf. Kasser and Sheldon, 2002).^6^

### Number of People That Participants Lived With and Saw on Thanksgiving

The sixth page of stimulus materials automatically piped in what the participants wrote on the first page. First, participants responded to the question, “How many other people did you see face-to-face on Thanksgiving Day **this year**?” using a slider that went from 0 to 100 in increments of 1 and showed the value. Next, participants responded to the question, “Including you, how many people normally live in your usual residence?” with response options of 1, 2, 3, 4, 5, 6, 7, or more (The average size of an American household in 2020 was 3.15 people; Statistica Research Department, 2021). The third question on this page was, “How many other people did you see face-to-face on Thanksgiving Day **last year, in 2019**?” to which participants responded using a slider that went from 0 to 100 in increments of 1 and showed the value. Finally, participants responded to the question, “Including you, how many people normally lived in your usual residence at that time last year?” with response options of 1, 2, 3, 4, 5, 6, 7, or more.

For our analyses that compared participants who did *versus* did not see anyone else face-to-face on Thanksgiving, we grouped participants into three categories. We based these categories on what research suggests may be important for assessing relationships between social contacts and wellbeing in the COVID-19 pandemic (Okabe-Miyamoto et al., 2021). They were for participants who reported seeing no one (*n*=27), one other person (*n*=63), or two or more other people (*n*=314) face-to-face on Thanksgiving.

### Pages After the Stimulus Materials

After the stimulus materials, participants reached two pages where they answered demographic questions and gave their impressions of the study. On the last page, participants received a debriefing to read and a code for them to show they had finished the study on Prolific.

## Results

Because of the large number of results, we provide most of the statistics in tables. We describe sensitivity power analyses in footnotes to make it easier to follow the main results.

### Descriptive Statistics and Correlations

Table 1 shows the correlations between the measures of regulatory focus, need satisfaction, affect, and satisfaction with Thanksgiving. It also shows the Cronbach’s alphas and descriptive statistics for these variables.^7^

### How Happy Were Participants on Thanksgiving?

Table 3 shows the percentage of participants scoring within different ranges of the three overall wellbeing variables in this study. This table shows that the holiday involved substantially more positive affect than negative affect for most participants, with 79.5% scoring above the scale midpoint on positive affect and 9.7% scoring above the midpoint on negative affect. Participants generally were satisfied with their holiday experience, with 64.9% scoring above the midpoint of satisfaction.

**Table 3:** Percentages of the sample scoring 1–2, 2–3, 3–4, 4–5, 5–6, and 6–7 on the three wellbeing variables during Thanksgiving.

Measure	% 1–2	% 2–3	% 3–4	% 4–5	% 5–6	% 6–7
Positive affect	5.2	4.7	10.6	15.9	33.4	30.2
Negative affect	56.9	19.8	13.6	5.5	2.5	1.7
Satisfaction	9.2	11.1	14.8	22.1	22.0	20.8

### Promotion and Prevention Focus on Thanksgiving

As shown in Table 4, participants reported being more prevention focused than promotion focused on Thanksgiving. We did three paired-samples *t*-tests between the regulatory focus indexes, with a Bonferroni adjustment of *p*=0.0167. Participants scored significantly lower on promotion (*M*=4.37, *SD*=1.32) than duties (*M*=5.22, *SD*=1.28) or caution/self-control (*M*=5.07, *SD*=1.38). With the Bonferroni adjustment, duties and caution/self-control did not differ significantly.^8^

**Table 4:** Tests of differences between measures of regulatory focus.

Measures in the test	*t*	*df*	Value of *p*	Mean diff.	*SD* diff.	95% CI	*d*
Duties *vs*. promotion	11.31	403	<0.001	0.85	1.51	[0.70, 1.00]	0.56
Caution/self-control *vs*. promotion	7.41	403	<0.001	0.69	1.88	[0.51, 0.88]	0.37
Duties *vs*. caution/self-control	1.97	403	0.049^a^	0.16	1.61	[0.00, 0.32]	0.10

a *With the Bonferroni adjustment of *p*=0.0167, this pair of means did not differ significantly*.

*Positive numbers indicate higher scores for the first variable in each pair. Mean diff. = mean of between-condition differences. SD diff. = standard deviation of between-condition differences. CI=confidence interval. d=Cohen’s d*.

### Regression Analyses on Affect and Satisfaction With Thanksgiving

We included each measure of regulatory focus and need satisfaction as simultaneous predictors of positive affect, negative affect, and satisfaction with Thanksgiving. Each multiple regression satisfied assumptions regarding multicollinearity, outliers, and normality of residuals. These results of the multiple regressions are in Table 5. According to G*Power, 404 participants provide 80% power to detect an individual coefficient in a six-predictor multiple regression with *f*^2^=0.02, *p*=0.05, two tailed, which is equivalent to *sr*^2^ of 0.0191.^9^ We emphasize results that met this criterion.

**Table 5:** Multiple regressions modeling relationships with positive affect, negative affect, and satisfaction with Thanksgiving.

Dependent variables and predictors	*B*	*β*	*sr* ^2^	Value of *p*	95% CI for *B*
Positive affect					
Autonomy	0.12	0.10	0.005	0.003	[0.04, 0.20]
Competence	−0.04	−0.03	<0.001	0.417	[−0.14, 0.06]
**Relatedness**	**0.43**	**0.37**	**0.061**	**<0.001**	**[0.35, 0.52]**
**Promotion**	**0.56**	**0.50**	**0.113**	**<0.001**	**[0.48, 0.64]**
Caution/self-control	−0.01	−0.01	<0.001	0.790	[−0.06, 0.05]
Duties	0.12	0.05	0.001	0.119	[−0.01, 0.12]
Negative affect					
Autonomy	−0.21	−0.19	0.019	<0.001	[−0.31, −0.11]
Competence	−0.13	−0.10	0.006	0.030	[−0.25, −0.01]
**Relatedness**	**−0.41**	**−0.40**	**0.070**	**<0.001**	**[−0.51, −0.30]**
Promotion	−0.16	−0.16	0.012	0.001	[−0.26, −0.06]
Caution/self-control	0.10	0.11	0.011	0.003	[0.04, 0.17]
Duties	0.03	0.03	<0.001	0.525	[−0.05, 0.11]
Satisfaction with Thanksgiving					
Autonomy	0.14	0.10	0.005	0.012	[0.03, 0.25]
Competence	−0.10	−0.06	0.002	0.154	[−0.23, 0.04]
**Relatedness**	**0.39**	**0.30**	**0.038**	**<0.001**	**[0.28, 0.51]**
**Promotion**	**0.67**	**0.53**	**0.124**	**<0.001**	**[0.56, 0.78]**
Caution/self-control	−0.09	−0.07	0.005	0.016	[−0.16, −0.02]
Duties	0.03	0.02	<0.001	0.516	[−0.06, 0.12]

*B, unstandardized regression weights. β, standardized regression weights. *sr*^2^, semi-partial correlation squared. CI, confidence interval. Rows in bold font indicate significant results with power>0.80*.

### Relationships With Positive Affect

Positive affect associated significantly and positively with promotion and relatedness, with *sr*^2^>0.0191. This is consistent with our hypotheses that promotion and relatedness satisfaction would associate positively with wellbeing. Additionally, positive affect associated significantly and positively to autonomy, but with *sr*^2^<0.0191.

### Relationships With Negative Affect

Negative affect associated significantly and negatively with relatedness with *sr*^2^>0.0191. This is consistent with our hypothesis that higher relatedness satisfaction would associate with more wellbeing. Additionally, negative affect associated significantly and negatively with promotion and competence, but with *sr*^2^<0.0191. Negative affect also associated significantly and positively with caution/self-control, but with *sr*^2^<0.0191.

### Relationships With Satisfaction With Thanksgiving

Satisfaction with Thanksgiving associated significantly and positively with promotion and relatedness, with *sr*^2^>0.0191. This is consistent with our hypothesis that promotion and relatedness satisfaction would associate positively with wellbeing. Satisfaction with Thanksgiving also associated significantly and positively with autonomy, but with *sr*^2^<0.0191. Additionally, satisfaction with Thanksgiving associated negatively with caution/self-control, but with *sr*^2^<0.0191.

### Analyses With Number of Face-to-Face Contacts

Participants reported seeing significantly fewer people face-to-face on Thanksgiving 2020 (*M*=5.14, *SD*=8.70) than on the previous Thanksgiving (*M*=13.99, *SD*=14.95), *t* (403)=−12.62, *p*<0.001, *d*=−0.63. This was not because of changes to the sizes of their usual households: Participants reported that about the same number of people lived in their normal residence on Thanksgiving 2020 (*M*=3.04, *SD*=1.46) as on the previous Thanksgiving (*M*=3.10, *SD*=1.45), *t* (403)=−1.11, *p*=0.268, *d*=−0.06. In this study, 78% of participants reported seeing fewer people on Thanksgiving in 2020 than on the previous Thanksgiving, 11.4% reported seeing the same number of people, and 7.7% of participants reported seeing more people on Thanksgiving 2020.

As expected, seeing anyone at Thanksgiving was associated with more wellbeing and relatedness satisfaction than seeing no one. We examined the other measures of need satisfaction and regulatory focus for exploratory purposes. Table 6 shows the results of one-way ANOVAs with Bonferroni-adjusted *post-hoc* comparisons. Those who saw either one or more people reported significantly higher relatedness satisfaction, promotion focus, positive affect, and satisfaction with Thanksgiving than participants who saw no one. The former two groups did not differ significantly on these variables.

**Table 6:** Tests of differences between participants who saw 0, 1, or 2+ other people face-to-face on Thanksgiving.

Measure and test	*df*s	*F*	Value of *p*	*η* ^2^	Mean diff.	Sig.	95% CI
**Autonomy**	**(2, 401)**	**9.37**	**<0.001**	**0.045**			
0 *vs*. 1 other					0.38	0.482	[−0.27, 1.04]
0 *vs*. 2+ others					−0.31	0.570	[−0.89, 0.26]
**1 *vs*. 2+ others**					**−0.70**	**<0.001**	**[−1.09, −0.30]**
Competence	(2, 401)	0.31	0.733	0.002			
0 *vs*. 1 other					0.17	1.000	[−0.36, 0.71]
0 *vs*. 2+ others					0.13	1.000	[−0.33, 0.60]
1 *vs*. 2+ others					−0.04	1.000	[−0.36, 0.28]
**Relatedness**	**(2, 401)**	**11.74**	**<0.001**	**0.055**			
**0 *vs*. 1 other**					**1.21**	**<0.001**	**[0.53, 1.89]**
**0 *vs*. 2+ others**					**1.18**	**<0.001**	**[0.59, 1.78]**
1 *vs*. 2+ others					−0.03	1.000	[−0.44, 0.38]
**Promotion**	**(2, 401)**	**7.90**	**<0.001**	**0.038**			
**0 *vs*. 1 other**					**1.05**	**0.001**	**[0.33, 1.76]**
**0 *vs*. 2+ others**					**1.02**	**<0.001**	**[0.40, 1.64]**
1 *vs*. 2+ others					−0.03	1.000	[−0.46, 0.40]
**Caution/self-control**	**(2, 401)**	**3.25**	**0.040**	**0.016**			
0 *vs*. 1 other					−0.26	1.000	[−1.02, 0.50]
0 *vs*. 2+ others					−0.58	0.108	[−1.24, 0.08]
1 *vs*. 2+ others					−0.32	0.286	[−0.77, 0.14]
**Duties**	**(2, 401)**	**22.30**	**<0.001**	**0.100**			
0 *vs*. 1 other					0.62	0.079	[−0.05, 1.30]
**0 *vs*. 2+ others**					**1.35**	**<0.001**	**[0.77, 1.94]**
**1 *vs*. 2+ others**					**0.73**	**<0.001**	**[0.33, 1.13]**
**Positive affect**	**(2, 401)**	**6.03**	**0.003**	**0.029**			
**0 *vs*. 1 other**					**0.96**	**0.013**	**[0.16, 1.76]**
**0 *vs*. 2+ others**					**1.01**	**0.002**	**[0.31, 1.71]**
1 *vs*. 2+ others					0.05	1.000	[−0.43, 0.53]
Negative affect	(2, 401)	2.30	0.102	0.011			
0 *vs*. 1 other					−0.57	0.167	[−1.28, 0.14]
0 *vs*. 2+ others					−0.54	0.106	[−1.16, 0.08]
1 *vs*. 2+ others					0.02	1.000	[−0.40, 0.45]
**Satisfaction with Thanksgiving**	**(2, 401)**	**10.80**	**<0.001**	**0.051**			
**0 *vs*. 1 other**					**1.51**	**<0.001**	**[0.62, 2.41]**
**0 *vs*. 2+ others**					**1.50**	**<0.001**	**[0.72, 2.28]**
1 *vs*. 2+ others					−0.01	1.000	[−0.55, 0.52]

*2+ others=saw two or more other people face-to-face. Group sizes: saw 0 other people (n=27), 1 other person (n=63), and 2+ others (n=314). Bonferroni post-hoc tests, in which numbers indicate higher means for the second condition within the pair. Mean diff. = mean of between-condition differences. CI=confidence interval. Bold font indicates rows with significant effects*.

Additionally, there were several other variables that showed significant between-group differences, and we report results of these exploratory analyses for the sake of completeness. Autonomy satisfaction was highest among those who saw one other person, and the other two groups did not differ significantly on this variable. A focus on duties and obligations was highest among those who saw two or more people, and the other two groups did not differ significantly on this variable. An ANOVA revealed a significant effect on caution/self-control, but *post-hoc* analyses revealed no significant differences between the three groups. The ANOVAs revealed no significant between-group differences on competence satisfaction or negative affect.

## Discussion

Several months into the COVID-19 pandemic, American participants reported being more prevention focused than promotion focused on Thanksgiving in 2020. However, most also reported having a happy and satisfying Thanksgiving, almost as much as on major holidays outside of the pandemic (e.g., Kasser and Sheldon, 2002). Participants had a happier and more satisfying Thanksgiving overall when they felt more connected with others and focused more on growth – that is, when they experienced more relatedness satisfaction and were more promotion focused. In contrast, associations of wellbeing with autonomy and competence satisfaction were weaker, as were associations of wellbeing with the duties and caution/self-control aspects of prevention focus. About 7% of participants in this study reported seeing no one face-to-face on Thanksgiving, compared with 11% of American respondents in a public opinion poll who saw no one face-to-face during the 2020 December holidays and 16% of respondents in that poll who saw no one face-to-face on the following New Year’s Eve and Day (Sanders, 2021). Wellbeing, relatedness satisfaction, and promotion focus were significantly higher among participants who saw anyone else face-to-face on Thanksgiving. These findings have implications for wellbeing at major holidays (not just in the pandemic), self-determination theory, and regulatory focus theory. Additionally, they have implications for persuasive messaging about how to gather safely at major holidays.

### Implications for Self-Determination Theory and Major Holidays

Major holidays emphasize maintaining and growing close relationships with family members (Fiese et al., 2002; Kasser and Sheldon, 2002; Páez et al., 2011; Allan et al., 2013; Hanke et al., 2016). Although satisfactions of autonomy, competence, and relatedness all are crucial for wellbeing (e.g., Deci and Ryan, 2000; Ryan and Deci, 2017), self-determination theory proposes that depending on the circumstances, one or another of the needs can “take the lead” in associating with wellbeing outcomes (Ryan and Deci, 2017, p. 247). The current research strengthens support for the idea that relatedness takes the lead in major holidays (also see Kasser and Sheldon, 2002; Páez et al., 2011; Allan et al., 2013; Hanke et al., 2016).

Additionally, we found that participants in the current study who reported seeing no one else face-to-face on Thanksgiving also reported lower relatedness satisfaction and wellbeing than participants who saw one or more people face-to-face that day. Consistent with research showing that living with a household partner but not the size of the household buffered negative effects of the pandemic on social connection (Okabe-Miyamoto et al., 2021), we found no significant differences in relatedness satisfaction or wellbeing between participants who saw one *versus* more people face-to-face on Thanksgiving. Of the 27 participants who saw no one else face-to-face, 85% reported seeing fewer people face-to-face on Thanksgiving 2020 than on the previous Thanksgiving. This finding suggests that these participants either could not find satisfying ways to connect with loved ones remotely, did not have anyone to reach out to that year, or had other things going on that day – such as quarantining – that could reduce relatedness satisfaction. A limitation of the current study is that it did not assess the specific ways participants connected with others remotely, and future research on wellbeing at major holidays in the pandemic could address this limitation. Public opinion polls commonly show that major holidays can be lonely times for people (Kerman, 2017; Savage, 2020), even when not in a pandemic, and future research on ways people may connect virtually with others could be important even once the pandemic is over.

### Implications for Regulatory Focus Theory and Major Holidays

During major holidays, including those not occurring in a pandemic, people can feel torn between ideally wanting to do something for the sake of enjoyment and feeling obligated to do something to maintain social relationships (e.g., Kasser and Sheldon, 2002; Allan et al., 2013; Hanke et al., 2016). Ideally wanting to do something is an aspect of promotion focus, whereas feeling obligated to do something is an aspect of prevention focus (e.g., Higgins, 1997; Vaughn et al., 2020). For example, an exciting opportunity to take a plane flight across the country to visit extended family can also feel obligatory if there are long lines and delays at the airport (The obligation to stand in long lines at airports also could conflict with another prevention goal, to remain safe against the virus, and to which we turn next.) Nonetheless, promotion was the regulatory focus that most strongly and consistently related to wellbeing: The more promotion-focused participants were on Thanksgiving, the happier and more satisfied they were with Thanksgiving Day. This expected positive relationship between promotion focus and wellbeing is consistent with other research on regulatory focus and wellbeing (e.g., Lanaj et al., 2012; Koopmann et al., 2016; Zhang et al., 2019; Wu and Chen, 2020).

Additionally, we speculate that during Thanksgiving in 2020, participants experienced a new prevention-prevention conflict specific to the pandemic. Participants’ descriptions of what they did that day, news accounts (Caspiani and Borter, 2020; Trotta and Layne, 2020), and the fact that duties and caution/self-control aspects of prevention focus loaded on different factors (cf., Haws et al., 2010; Higgins et al., 2001; Vaughn et al., 2020) suggest that many people experienced conflicts between two types of prevention goals. It appears that one was to fulfill duties to maintain holiday traditions by gathering with extended family. The other was to remain cautious and exert self-control to protect the self and others from COVID-19.

Even so, being focused on duties at Thanksgiving in 2020 did not relate significantly to any wellbeing measures, when controlling for relationships between measures of need satisfaction and regulatory focus. Additionally, being focused on caution/self-control at Thanksgiving 2020 related to less wellbeing, but even when these associations were statistically significant, they were weak. Thus, in contrast to what other research has shown about relationships between prevention focus and affect and/or wellbeing (e.g., Lanaj et al., 2012; Koopmann et al., 2016; Zhang et al., 2019; Wu and Chen, 2020), prevention focus on Thanksgiving 2020 did not relate strongly to lower wellbeing. While obligations can feel pressuring (e.g., Chen et al., 2015; Vaughn, 2018), they can be highly meaningful at holidays (e.g., Hanke et al., 2016). Many Americans had not seen extended family for months before Thanksgiving 2020 (Caspiani and Borter, 2020; Trotta and Layne, 2020), which could have made fulfilling duties to maintain in-person Thanksgiving traditions feel more meaningful than usual, even when balanced against remaining careful. Future research could examine whether the often-negative relationships between prevention focus and wellbeing (cf. Grant and Higgins, 2003; Lanaj et al., 2012; Koopmann et al., 2016; Zhang et al., 2019; Wu and Chen, 2020) generally are weaker during major holidays.

### Implications for Persuasive Messaging About COVID-19 at Major Holidays

The strongest predictors of wellbeing at Thanksgiving in this study were feeling connected with others and focusing on growth – that is, relatedness satisfaction and promotion focus. Given that promotion focus serves the survival needs for growth and nurturance (Higgins, 1997, 1998), the current findings suggest that persuasive communications that support wellbeing at major holidays should emphasize ways to nurture connections with close others. Vigilance is effortful and stressful (Warm et al., 2008), but emphasizing relatedness support and promotion focus on a holiday - while also remaining safe – could help balance the strong vigilance involved in preventing the spread of the virus. The tension between having an ideal holiday celebration and maintaining vigilance against the virus may not go away while the pandemic continues. However, research on how regulatory fit can affect persuasion suggests that this tension could be useful.

Regulatory fit (Higgins, 2000) occurs when people think about or use strategies for pursuing a goal (e.g., making sure everything goes right at the holiday celebration) that fit and sustain their regulatory focus toward the goal (e.g., having the ideal holiday celebration). Regulatory nonfit occurs when people think about or strategies for pursuing a goal (e.g., being careful and making sure nothing goes wrong) that do not fit or sustain their regulatory focus toward the goal (e.g., having the ideal holiday celebration). Regulatory fit feels right (Higgins, 2000, 2005) and people can attribute this feeling of rightness to what they are judging (e.g., for reviews, see Higgins, 2000; Higgins, 2005; Vaughn et al., 2006a,b, 2010b; Avnet and Higgins, 2021). They may assume that if they feel right when thinking about something (e.g., ways to have the ideal holiday celebration), it is because what they are thinking about is right. Regulatory fit can enhance persuasion through advocacy messages, which explicitly intend to persuade (e.g., Cesario et al., 2004, 2008; Lee and Aaker, 2004; Koenig et al., 2009; Ludolph and Schulz, 2015), and through narratives, in which the explicit intent is often more subtle (Vaughn et al., 2009, 2010a).

With that said, when people’s initial attitudes about a topic are strongly negative, regulatory nonfit can de-intensify negative attitudes and increase how carefully people think about a message that opposes their initial attitudes (Fridman et al., 2016, 2018a,b). This upside of regulatory nonfit (Avnet and Higgins, 2021) could be useful in communications about how to have a good holiday with others while remaining safe against COVID-19. Ideally wanting to have a better holiday than one’s dreary pandemic norm could be a promotion-focused goal, whereas being careful and exerting self-control against the virus could be examples of vigilant strategies of goal pursuit. Research on regulatory nonfit (Avnet and Higgins, 2021) suggests that messages that include regulatory nonfit could lead people to think more abstractly and creatively about how to have a good holiday. Such messages could have especially positive impacts on people who initially have the strongest attitudes (Fridman et al., 2016) and are determined to have a holiday gathering that lives up to their ideals, no matter how risky. Indeed, many organizations’ suggestions about how to celebrate holidays safely during the COVID-19 pandemic have incorporated this regulatory nonfit implicitly (e.g., Poplett, 2020; Centers for Disease Control and Prevention, 2021). Future research could examine whether, how, and for whom the upsides of regulatory nonfit extend to communications about how to have safe and enjoyable holiday celebrations in a pandemic. This future research could examine both wellbeing and physical health, including the percentages of participants who contract COVID-19 during the holiday. Such research would likely also be relevant to future epidemics and pandemics, which may become more frequent (Hilsenrath, 2020).

### Limitations and Future Research

Although we speculate that participants in this study experienced goal conflict at Thanksgiving, this study had no measures of goal conflict (cf., Boudreaux and Ozer, 2013; Gere and Schimmack, 2013; Gray et al., 2017; Vowels and Carnelley, 2020). Goal conflict occurs when pursuing one valued goal interferes with the pursuit of another valued goal. Wellbeing relates negatively to goal conflict, either when the conflict is between one’s own goals (e.g., Boudreaux and Ozer, 2013; Gray et al., 2017) or between the goals of relationship partners (e.g., Gere and Schimmack, 2013; Vowels and Carnelley, 2020). Conflicts between the goal of gathering face-to-face and the goal of remaining safe from COVID-19 will likely continue for the duration of the pandemic. Research on goal conflict and wellbeing in the pandemic could examine how people manage these conflicting goals on major holidays and at other times.

The present research was cross-sectional, so we cannot infer causality from the results. Although we treated positive affect, negative affect, and satisfaction with Thanksgiving as outcome variables, they could themselves be predictors of the other variables in the study. Moreover, future research could measure participants’ baseline wellbeing to understand how it relates to happiness and other aspects of wellbeing at Thanksgiving. Future research could use a diary study design to address these limitations (e.g., Allan et al., 2013).

Another limitation of this research is that it did not have a representative sample of Americans. Prolific samples resemble MTurk samples (Peer et al., 2017), and MTurk samples do not represent the general US population (Goodman et al., 2013; Walters et al., 2018). For example, MTurk samples tend to be younger, more educated, less employed, and have more White and Asian respondents and fewer Black and Latinx or Hispanic respondents than the general US population (Walters et al., 2018). COVID-19 has stronger impacts on people who are older (McCarthy, 2020) and on people of color (Centers for Disease Control and Prevention, 2020a), which could have affected wellbeing at Thanksgiving.

Future cross-cultural research on social connection, regulatory focus, and wellbeing at major holidays in a pandemic could find stronger relationships between prevention focus and wellbeing. People tend to be somewhat promotion oriented in contexts that emphasize the values of individualism, such as the United States (e.g., Lee et al., 2000). In more collectivist cultural contexts that emphasize the value of fulfilling duties and obligations (e.g., Miller et al., 2011; Buchtel et al., 2018), people who believe they are maintaining important holiday traditions may have substantially higher wellbeing. Additionally, prevention focus may be a better fit for cultural contexts with tighter norms, whereas promotion focus may be a better fit for cultures with looser norms (Kumar et al., 2019), such as the United States (Gelfand et al., 2011). In contexts with stronger norms and less tolerance for deviant behavior, the prevention focus on minimizing losses could be especially valued and meaningful – especially if it means protecting loved ones from COVID-19 on a major holiday.

The current study was designed to be basic research on how need satisfaction and regulatory focus relate to wellbeing in a specific context, and as such, it was designed to extend theory and research in three areas: wellbeing on major holidays, self-determination theory, and regulatory focus theory. Given that numerous other studies have examined wellbeing and mental health in the COVID-19 pandemic (e.g., Dawel et al., 2020; Gray et al., 2020; Mental Health America, 2020; Vanderweele et al., 2020; National Center for Health Statistics, 2021; Okabe-Miyamoto and Lyubomirsky, 2021; Panchal et al., 2021), the current study’s unique emphasis on wellbeing during a major holiday in the pandemic is both a strength and a limitation. We did not design this study to assess aspects of mental health, such as depression or anxiety, and it is beyond the scope of the current research to speculate at length about implications of our findings for these aspects of mental health. However, we hope that the current work inspires future researchers to apply these findings to design and rigorously test interventions that may protect or improve mental health in the COVID-19 pandemic and beyond.

## Conclusion

In 2020, American Thanksgiving occurred 8months into the COVID-19 pandemic, when many people had become more stressed, anxious, depressed, and lonely (e.g., Mental Health America, 2020; National Center for Health Statistics, 2021; Ravenscraft, 2021). Participants reported being more prevention focused than promotion focused on Thanksgiving in 2020. However, most also reported having a happy and satisfying Thanksgiving. Participants had a happier Thanksgiving overall when they felt more connected with others and focused more on growth. While these experiences could be especially important during major holidays in a pandemic, they could be just as important just as important once the pandemic is over, on any day when simultaneously having fun and meeting obligations is important.

Overall, this study suggests that messages about how to accomplish one’s ideals and connect with others at holidays could help boost wellbeing. Additionally, research suggests that it could be beneficial to include information about how to remain safe against the virus, especially for people who have the strongest intentions to gather with many people who might not be vaccinated (see Fridman et al., 2016). We hope future research will explore this possibility, both for the COVID-19 pandemic and for any future epidemics.

## Data Availability Statement

The datasets presented in this study can be found in online repositories. The names of the repository/repositories and accession number(s) can be found at: Open Science Framework, https://osf.io/fy6rc/.

## Ethics Statement

The studies involving human participants were reviewed and approved by Ithaca College Institutional Review Board. The participants provided their written informed consent to participate in this study.

## Author Contributions

LV conceptualized and designed the research, acquired the data, and wrote the first draft of the manuscript. LV, PB, RC, JJ, and CG contributed to the analysis and interpretation of the data. LV, PB, RC, JJ, CG, and JL commented on the previous versions of the manuscript, and read and approved the final manuscript. RC, JJ, and CG contributed equally, and their authorship order was decided by random draw. All authors contributed to the article and approved the submitted version.

## Conflict of Interest

JL was employed by Confluent Sciences, LLC. He is now employed by the Air Force Office of Scientific Research.

The remaining authors declare that the research was conducted in the absence of any commercial or financial relationships that could be construed as a potential conflict of interest.

## Publisher’s Note

All claims expressed in this article are solely those of the authors and do not necessarily represent those of their affiliated organizations, or those of the publisher, the editors and the reviewers. Any product that may be evaluated in this article, or claim that may be made by its manufacturer, is not guaranteed or endorsed by the publisher.

## Footnotes

^1^Relationships between each regulatory focus and satisfaction of needs for autonomy, competence, and relatedness could differ depending on the context. These are important relationships to examine because they pertain to the need-support model (Vaughn, 2017), which bridges self-determination theory and regulatory focus theory. Thus, although exploring relationships between regulatory focus and need satisfaction were not the main goal of the current research, Supplementary Table S1, S2 show results of the analyses of these relationships. In one set of analyses, we treated promotion and prevention focus as simultaneous predictors of each type of need satisfaction. In the other set of analyses, we treated autonomy, competence, and relatedness satisfaction as simultaneous predictors of promotion and prevention focus. Overall, we found strong, positive relationships between promotion focus and satisfaction of autonomy and relatedness. Relationships with need satisfaction differed substantially between duties and caution/self-control aspects of prevention focus. Consistent with common views of duties as pressuring (e.g., Deci and Ryan, 2000; Chen et al., 2015; Ryan and Deci, 2017; Vansteenkiste et al., 2020), participants who reported being more dutiful at Thanksgiving reported feeling less able to do what they wanted and more pressured on that day – that is, less autonomy satisfied. However, they also felt more competent and connected to others. In contrast, focusing on caution and self-control at Thanksgiving related only weakly to any type of need satisfaction. Caution/self-control may often be for maintaining the status quo, and as such, it could be somewhat boring (Ecker and Gilead, 2018) – especially on a celebratory day like Thanksgiving.

^2^No state had a majority of participants. California had the most with 41 participants (10.1%), followed by New York with 38 (9.4%), Illinois with 21 (5.2%), and Michigan with 19 (4.7%).

^3^This page also contained the four-item Beneficence Scale (Martela and Ryan, 2016), which we included for exploratory purposes. It includes statements such as, “I felt that my actions had a positive impact on the people around me,” and “The things I did contributed to the betterment of society.” The Supplementary Material contains the results of all analyses with the Beneficence Scale included.

^4^G*Power does not include factor analysis. However, 200 participants support an exploratory factor analysis under moderately good conditions (communalities of 0.40 to 0.70, with at least three measured variables per theorized factor; Fabrigar and Wegner, 2012). Several of the communalities in our analyses were slightly below this range, and 400 participants can support an exploratory factor analysis under these conditions (Fabrigar and Wegner, 2012).

^5^Because promotion focus and positive affect were strongly correlated (*r* = 0.82), we submitted the items in these two scales to an exploratory factor analysis using maximum likelihood estimation and direct oblimin rotation, with delta = 0. The Kaiser-Meyer-Olkin measure showed that the sampling was adequate, KMO = 0.958, and Bartlett’s test χ^2^ (120) = 4597.31, *p* < 0.001. Table S3 in the Online Resource shows the pattern-matrix factor loadings and the communalities for the items. The analysis revealed two factors, one for promotion focus and the other for positive affect. Together, these factors together accounted for 66.31% of the variance, and they correlated at *r* = 0.79. The only item that loaded >0.40 on both factors was “Being optimistic.” Given the importance of optimism in supporting eager goal pursuit (e.g., Grant and Higgins, 2003; Ouschan et al., 2007; Hazlett et al., 2011), we retained the optimism item in the promotion focus scale.

^6^Because promotion focus and satisfaction with Thanksgiving were strongly correlated (*r* = 0.78), we submitted the items in these two scales to an exploratory factor analysis using maximum likelihood estimation and direct oblimin rotation, with delta = 0. The Kaiser-Meyer-Olkin measure showed that the sampling was adequate, KMO = 0.938, and Bartlett’s test χ^2^ (66) = 3483.26, *p* < 0.001. Table S4 in the Online Resource shows the pattern-matrix factor loadings and the communalities for the items. The analysis revealed two factors, one for promotion focus and the other for satisfaction with Thanksgiving. Together, these factors accounted for 60.77% of the variance, and they correlated at *r* = −0.81. The only unexpected loading was for “I did what I ideally liked to,” which loaded >0.40 on the satisfaction with Thanksgiving factor. Given the importance of ideals in promotion focus (e.g., Higgins, 1997, 1998; Molden et al., 2007; Scholer et al., 2019a), we retained the ideals item in the promotion focus scale.

^7^We did sensitivity power analyses for bivariate normal correlations. According to G*Power, 404 participants provide 80% power to detect a Pearson *r* of 0.14, *p* = 0.05, two tailed.

^8^According to G*Power, 404 participants provide 80% power to detect a difference in a paired-samples t-test of *d* = 0.16, *p* = 0.0167, two tailed.

^9^We did a sensitivity power analysis for a single regression coefficient in a six-predictor linear multiple regression with 404 participants. G*Power provides *f*^2^s for this type of analysis. In our multiple regressions, we used *sr*^2^ as the measure of effect size, as recommended by Disabato (2016). The variable, *sr*^2^, is the correlation between the predictor of interest and the dependent variable, controlling for the relationships between the other predictors and the predictor of interest. To translate between *f*^2^ and *sr*^2^, we used an online calculator (Lenhard and Lenhard, 2016) to determine the correlation *r*s that were equivalent to the square roots of the *f*^2^s, and we squared those *r*s. According to G*Power, 404 participants provide 80% power to detect an individual coefficient in a six-predictor multiple regression with *f*^2^ = 0.0195, *p* = 0.05, two tailed, which is equivalent to *sr*^2^ of 0.0191. This is the same as what G*Power shows for three-predictor, four-predictor, and seven-predictor multiple regressions, which is relevant to exploratory analyses contained in Supplementary Table S1, S2. These include analyses predicting regulatory focus from need satisfaction and *vice-versa*, as well as all regression analyses including the candidate need for beneficence.

## References

[ref1] Abidi S. Gramlich J. (2020). As CDC warned against holiday travel, 57% of Americans say they changed Thanksgiving plans due to COVID-19. Pew Research Center. Available at: https://www.pewresearch.org/fact-tank/2020/12/22/as-cdc-warned-against-holiday-travel-57-of-americans-say-they-changed-thanksgiving-plans-due-to-covid-19/ (Accessed December 22, 2020).

[ref2] Allan B. A. Steger M. F. Shin J. Y. (2013). Thanks? Gratitude and well-being over the thanksgiving holiday among college students. J. Posit. Psychol. 8, 91–102. doi: 10.1080/17439760.2013.776623

[ref3] Avnet T. Higgins E. T. (2021). Regulatory fit and non-fit: how they work and what they do. SSRN [Preprint]. doi: 10.2139/SSRN.3824009

[ref4] Baumeister R. F. Leary M. R. (1995). The need to belong: desire for interpersonal attachments as a fundamental human motivation. Psychol. Bull. 117, 497–529. doi: 10.1037/0033-2909.117.3.497, PMID: 7777651

[ref6] Boudreaux M. J. Ozer D. J. (2013). Goal conflict, goal striving, and psychological well-being. Motiv. Emot. 37, 433–443. doi: 10.1007/s11031-012-9333-2

[ref7] Buchtel E. E. Ng L. C. Y. Norenzayan A. Heine S. J. Biesanz J. C. Chen S. X.. (2018). A sense of obligation: cultural differences in the experience of obligation. Personal. Soc. Psychol. Bull. 44, 1545–1566. doi: 10.1177/0146167218769610, PMID: 29742994

[ref8] Caspiani M. Borter G. (2020). Americans defy pandemic, political leaders to travel for Thanksgiving. Reuters. Available at: https://www.reuters.com/article/health-coronavirus-usa/americans-defy-pandemic-political-leaders-to-travel-for-thanksgiving-idUSKBN2851R6 (Accessed November 25, 2020).

[ref9] Centers for Disease Control and Prevention (2020a). COVID-19 in racial and ethnic minority groups. Available at: https://www.cdc.gov/coronavirus/2019-ncov/need-extra-precautions/racial-ethnic-minorities.html. (Accessed June 25, 2020).

[ref10] Centers for Disease Control and Prevention (2020b). Get your home ready. Available at: https://www.cdc.gov/coronavirus/2019-ncov/daily-life-coping/get-your-household-ready-for-COVID-19.html (Accessed March 27, 2020).

[ref11] Centers for Disease Control and Prevention (2020c). Social distancing, quarantine, and isolation. Available at: https://www.cdc.gov/coronavirus/2019-ncov/prevent-getting-sick/social-distancing.html (Accessed July 15, 2020).

[ref12] Centers for Disease Control and Prevention (2021). Holidays. Available at: https://www.cdc.gov/coronavirus/2019-ncov/daily-life-coping/holidays/winter.html (Accessed April 21, 2021).

[ref13] Cesario J. Grant H. Higgins E. T. (2004). Regulatory fit and persuasion: transfer from “feeling right”. J. Pers. Soc. Psychol. 86, 388–404. doi: 10.1037/0022-3514.86.3.388, PMID: 15008644

[ref14] Cesario J. Higgins E. T. Scholer A. A. (2008). Regulatory fit and persuasion: basic principles and remaining questions. Soc. Personal. Psychol. Compass 2, 444–463. doi: 10.1111/j.1751-9004.2007.00055.x

[ref15] Chen B. Vansteenkiste M. Beyers W. Boone L. Deci E. L. Van der Kaap-Deeder J.. (2015). Basic psychological need satisfaction, need frustration, and need strength across four cultures. Motiv. Emot. 39, 216–236. doi: 10.1007/s11031-014-9450-1

[ref17] Dawel A. Shou Y. Smithson M. Cherbuin N. Banfield M. Calear A. L.. (2020). The effect of COVID-19 on mental health and wellbeing in a representative sample of Australian adults. Front. Psych. 11:1026. doi: 10.3389/fpsyt.2020.579985, PMID: 33132940 PMC7573356

[ref18] Deci E. L. Ryan R. M. (2000). The “what” and “why” of goal pursuits: human needs and the self-determination of behavior. Psychol. Inq. 11, 227–268. doi: 10.1207/S15327965PLI1104_01

[ref19] Diener E. Wirtz D. Tov W. Kim-Prieto C. Choi D. Oishi S.. (2010). New well-being measures: short scales to assess flourishing and positive and negative feelings. Soc. Indic. Res. 97, 143–156. doi: 10.1007/s11205-009-9493-y

[ref20] Disabato D. (2016). On effect sizes in multiple regression. Available at: http://www.daviddisabato.com/blog/2016/4/8/on-effect-sizes-in-multiple-regression (Accessed April 8, 2016).

[ref21] Ecker Y. Gilead M. (2018). Goal-directed allostasis: the unique challenge of keeping things as they are and strategies to overcome it. Perspect. Psychol. Sci. 13, 618–633. doi: 10.1177/1745691618769847, PMID: 30040911

[ref22] Fabrigar L. R. Wegener D. T. (2012). Exploratory Factor Analysis Oxford University Press.

[ref23] Faul F. Erdfelder E. Lang A.-G. Buchner A. (2007). G*Power 3: a flexible statistical power analysis program for the social, behavioral, and biomedical sciences. Behav. Res. Methods 39, 175–191. doi: 10.3758/BF03193146, PMID: 17695343

[ref24] Fay D. Urbach T. Scheithauer L. (2019). What motivates you right now? Development of a measure of momentary-chronic regulatory focus. Meas. Instrum. Social Sci. 2:5. doi: 10.1186/s42409-019-0007-7

[ref25] Fiese B. H. Tomcho T. J. Douglas M. Josephs K. Poltrock S. Baker T. (2002). A review of 50 years of research on naturally occurring family routines and rituals: cause for celebration? J. Fam. Psychol. 16, 381–390. doi: 10.1037/0893-3200.16.4.381, PMID: 12561283

[ref27] Fridman I. Glare P. A. Stabler S. M. Epstein A. S. Wiesenthal A. Leblanc T. W.. (2018a). Information framing reduces initial negative attitudes in cancer patients’ decisions about hospice care. J. Pain Symptom Manag. 55, 1540–1545. doi: 10.1016/j.jpainsymman.2018.02.010, PMID: 29474940 PMC8725201

[ref28] Fridman I. Scherr K. A. Glare P. A. Higgins E. T. (2016). Using a non-fit message helps to de-intensify negative reactions to tough advice. Personal. Soc. Psychol. Bull. 42, 1025–1044. doi: 10.1177/0146167216649931, PMID: 27341845 PMC5610136

[ref29] Fridman I. Ubel P. A. Higgins E. T. (2018b). Eye-tracking evidence shows that non-fit messaging impacts attention, attitudes and choice. PLoS One 13:e0205993. doi: 10.1371/journal.pone.0205993, PMID: 30365559 PMC6203368

[ref30] Gelfand M. J. Raver J. L. Nishii L. Leslie L. M. Lun J. Lim B. C.. (2011). Differences between tight and loose cultures: a 33-nation study. Science 332, 1100–1104. doi: 10.1126/science.1197754, PMID: 21617077

[ref31] Gere J. Schimmack U. (2013). When romantic partners’ goals conflict: effects on relationship quality and subjective well-being. J. Happiness Stud. 14, 37–49. doi: 10.1007/s10902-011-9314-2

[ref32] Gilbert D. Abdullah J. (2004). Holidaytaking and the sense of well-being. Ann. Tour. Res. 31, 103–121. doi: 10.1016/j.annals.2003.06.001

[ref33] Goodman J. K. Cryder C. E. Cheema A. (2013). Data collection in a flat world: the strengths and weaknesses of Mechanical Turk samples. J. Behav. Decis. Mak. 26, 213–224. doi: 10.1002/bdm.1753

[ref34] Grant H. Higgins E. T. (2003). Optimism, promotion pride, and prevention pride as predictors of quality of life. Personal. Soc. Psychol. Bull. 29, 1521–1532. doi: 10.1177/0146167203256919, PMID: 15018683

[ref35] Grantham-Philips W. (2020). Ready for Thanksgiving 2020? Make sure you do these 8 things before Thursday. USA Today. Available at: https://www.usatoday.com/story/news/nation/2020/11/23/thanksgiving-2020-make-sure-you-do-these-8-things-before-thursday/6392082002/ (Accessed November 23, 2020).

[ref36] Gray N. S. O’Connor C. Knowles J. Pink J. Simkiss N. J. Williams S. D.. (2020). The influence of the COVID-19 pandemic on mental well-being and psychological distress: impact upon a single country. Front. Psych. 11:594115. doi: 10.3389/fpsyt.2020.594115, PMID: 33262714 PMC7686842

[ref37] Gray J. S. Ozer D. J. Rosenthal R. (2017). Goal conflict and psychological well-being: a meta-analysis. J. Res. Pers. 66, 27–37. doi: 10.1016/j.jrp.2016.12.003

[ref38] Hanke K. van Egmond M. C. Crespo C. Boer D. (2016). Blessing or burden? The role of appraisal for family rituals and flourishing among LGBT adults. J. Fam. Psychol. 30, 562–568. doi: 10.1037/fam0000214, PMID: 27159796

[ref40] Haws K. L. Dholakia U. M. Bearden W. O. (2010). An assessment of chronic regulatory focus measures. J. Mark. Res. 47, 967–982. doi: 10.1509/jmkr.47.5.967

[ref41] Hazlett A. Molden D. C. Sackett A. M. (2011). Hoping for the best or preparing for the worst? regulatory focus and preferences for optimism and pessimism in predicting personal outcomes. Soc. Cogn. 29, 74–96. doi: 10.1521/soco.2011.29.1.74

[ref42] Higgins E. T. (1997). Beyond pleasure and pain. Am. Psychol. 52, 1280–1300. doi: 10.1037/0003-066X.52.12.1280, PMID: 9414606

[ref43] Higgins E. T. (1998). “Promotion and prevention: regulatory focus as a motivational principle,” in Advances in Experimental Social Psychology. *Vol*. 30. ed. Zanna M. P. (New York: Academic Press), 1–46.

[ref44] Higgins E. T. (2000). Making a good decision: value from fit. Am. Psychol. 55, 1217–1230. doi: 10.1037/0003-066X.55.11.1217, PMID: 11280936

[ref45] Higgins E. T. (2005). Value from regulatory fit. Curr. Dir. Psychol. Sci. 14, 209–213. doi: 10.1111/j.0963-7214.2005.00366.x

[ref46] Higgins E. T. Friedman R. S. Harlow R. E. Idson L. C. Ayduk O. N. Taylor A. (2001). Achievement orientations from subjective histories of success: promotion pride versus prevention pride. Eur. J. Soc. Psychol. 31, 3–23. doi: 10.1002/ejsp.27

[ref47] Hilsenrath J. (2020). Global virus outbreaks like coronavirus, once rare, will become more common. Wall Street Journal. Available at: https://www.wsj.com/articles/viral-outbreaks-once-rare-become-part-of-the-global-landscape-11583455309 (Accessed March 6, 2020).

[ref48] Hodis F. A. (2017). Investigating the structure of regulatory focus: a bifactor analysis. Personal. Individ. Differ. 109, 192–200. doi: 10.1016/j.paid.2017.01.004

[ref49] Idson L. C. Liberman N. Higgins E. T. (2000). Distinguishing gains from nonlosses and losses from nongains: a regulatory focus perspective on hedonic intensity. J. Exp. Soc. Psychol. 36, 252–274. doi: 10.1006/jesp.1999.1402

[ref50] JASP Team (2020). JASP (Version 0.13.1) [Computer software].

[ref51] Kasser T. Sheldon K. M. (2002). What makes for a merry Christmas? J. Happiness Stud. 3, 313–329. doi: 10.1023/A:1021516410457

[ref52] Kerman S. (2017). The holiday season: joy, love and loneliness. AARP Res. doi: 10.26419/res.00184.001

[ref53] Kim J. Chen K. Davis W. E. Hicks J. A. Schlegel R. J. (2019). Approaching the true self: promotion focus predicts the experience of authenticity. J. Res. Pers. 78, 165–176. doi: 10.1016/j.jrp.2018.12.001

[ref54] Knee C. R. Hadden B. W. Porter B. Rodriguez L. M. (2013). Self-determination theory and romantic relationship processes. Personal. Soc. Psychol. Rev. 17, 307–324. doi: 10.1177/1088868313498000, PMID: 23921674

[ref55] Koenig A. M. Cesario J. Molden D. C. Kosloff S. Higgins E. T. (2009). Incidental experiences of regulatory fit and the processing of persuasive appeals. Personal. Soc. Psychol. Bull. 35, 1342–1355. doi: 10.1177/0146167209339076, PMID: 19571272

[ref57] Koopmann J. Lanaj K. Bono J. Campana K. (2016). Daily shifts in regulatory focus: the influence of work events and implications for employee well-being. J. Organ. Behav. 37, 1293–1316. doi: 10.1002/job.2105

[ref58] Kumar R. Budhwar P. Patel C. Varma A. (2019). Self-regulation and expatriate adjustment: the role of regulatory fit. Hum. Resour. Manag. Rev. 29:100666. doi: 10.1016/j.hrmr.2018.09.002

[ref59] Lanaj K. Chang C.-H. Johnson R. E. (2012). Regulatory focus and work-related outcomes: a review and meta-analysis. Psychol. Bull. 138, 998–1034. doi: 10.1037/a0027723, PMID: 22468880

[ref60] Lee A. Y. Aaker J. L. (2004). Bringing the frame into focus: the influence of regulatory fit on processing fluency and persuasion. J. Pers. Soc. Psychol. 86, 205–218. doi: 10.1037/0022-3514.86.2.205, PMID: 14769079

[ref61] Lee A. Y. Aaker J. L. Gardner W. L. (2000). The pleasures and pains of distinct self-construals: the role of interdependence in regulatory focus. J. Pers. Soc. Psychol. 78, 1122–1134. doi: 10.1037/0022-3514.78.6.1122, PMID: 10870913

[ref62] Lenhard W. Lenhard A. (2016). Calculation of Effect Sizes. Dettelbach, Germany: Psychometrica.

[ref63] Lockwood P. Jordan C. H. Kunda Z. (2002). Motivation by positive or negative role models: regulatory focus determines who will best inspire us. J. Pers. Soc. Psychol. 83, 854–864. doi: 10.1037/0022-3514.83.4.854, PMID: 12374440

[ref64] Ludolph R. Schulz P. J. (2015). Does regulatory fit lead to more effective health communication? A systematic review. Soc. Sci. Med. 128, 142–150. doi: 10.1016/j.socscimed.2015.01.021, PMID: 25617673

[ref65] Manczak E. M. Zapata-Geitl C. McAdams D. P. (2014). Regulatory focus in the life story: prevention and promotion as expressed in three layers of personality. J. Pers. Soc. Psychol. 106, 169–181. doi: 10.1037/a0034951, PMID: 24377362

[ref66] Martela F. Ryan R. M. (2016). The benefits of benevolence: basic psychological needs. Beneficence, and the enhancement of well-being. J. Pers. 84, 750–764. doi: 10.1111/jopy.12215, PMID: 26249135

[ref67] McCarthy J. (2015). Holidays, weekends still Americans' happiest days of year. Gallup. Available at: https://news.gallup.com/poll/180911/holidays-weekends-americans-happiest-days-year.aspx (Accessed January 13, 2015).

[ref68] McCarthy N. (2020). How COVID-19 affects different U.S. age groups. Statistica. Available at: https://www.statista.com/chart/21173/hospitalization-icu-admission-and-fatality-rates-for-reported-coronavirus-cases/ (Accessed March 19, 2020).

[ref69] Mental Health America (2020). COVID-19 and mental health: A growing crisis. Available at: https://www.mhanational.org/research-reports/covid-19-and-mental-health-growing-crisis (Accessed October 20, 2020).

[ref70] Miller J. G. Das R. Chakravarthy S. (2011). Culture and role of choice in agency. J. Pers. Soc. Psychol. 101, 46–61. doi: 10.1037/a0023330, PMID: 21480735

[ref71] Milyavskaya M. Gingras I. Mageau G. A. Koestner R. Gagnon H. Fang J.. (2009). Balance across contexts: importance of balanced need satisfaction across various life domains. Personal. Soc. Psychol. Bull. 35, 1031–1045. doi: 10.1177/0146167209337036, PMID: 19592677

[ref72] Molden D. C. Lee A. Higgins E. T. (2007). “Motivation for promotion and prevention” in Handbook of Motivation Science. eds. J. Shah W. Gardner, New York: Guilford Press, 101–128.

[ref73] National Center for Health Statistics (2021). Anxiety and depression: household pulse survey. Centers for Disease Control and Prevention. Available at: https://www.cdc.gov/nchs/covid19/pulse/mental-health.htm (Accessed January 6, 2021).

[ref74] Nawijn J. Marchand M. A. Veenhoven R. Vingerhoets A. J. (2010). Vacationers happier, but most not happier after a holiday. Appl. Res. Qual. Life 5, 35–47. doi: 10.1007/s11482-009-9091-9, PMID: 20234864 PMC2837207

[ref75] Okabe-Miyamoto K. Folk D. Lyubomirsky S. Dunn E. W. (2021). Changes in social connection during COVID-19 social distancing: It’s not (household) size that matters, it's who you're with. PLoS One 16:e0245009. doi: 10.1371/journal.pone.0245009, PMID: 33471811 PMC7817035

[ref76] Okabe-Miyamoto K. Lyubomirsky S. (2021). “Social connection and well-being during COVID-19,” in *World Happiness Report*, 2021. eds. Helliwell J. F. Layard R. Sachs J. D. De Neve J. E. Aknin L. B. Wang S., New York: Sustainable Development Solutions Network, 131–152.

[ref77] Ouschan L. Boldero J. M. Kashima Y. Wakimoto R. Kashima E. S. (2007). Regulatory focus strategies scale: a measure of individual differences in the endorsement of regulatory strategies. Asian J. Soc. Psychol. 10, 243–257. doi: 10.1111/j.1467-839X.2007.00233.x

[ref78] Páez D. M. Bilbao Á. Bobowik M. Campos M. Basabe N. (2011). Merry Christmas and happy new year! The impact of Christmas rituals on subjective well-being and family’s emotional climate. Revista de Psicología Social 26, 373–386. doi: 10.1174/021347411797361347

[ref79] Palan S. Schitter S. (2018). Prolific.Ac—A subject pool for online experiments. J. Behav. Exp. Financ. 17, 22–27. doi: 10.1016/j.jbef.2017.12.004

[ref80] Panchal N. Kamal R. Cox C. Garfield R. (2021). The implications of COVID-19 for mental health and substance use. KFF. Available at: https://www.kff.org/coronavirus-covid-19/issue-brief/the-implications-of-covid-19-for-mental-health-and-substance-use/ (Accessed February 10, 2021).

[ref81] Pavot W. Diener E. (1993). Review of the satisfaction with life scale. Psychol. Assess. 5, 164–172. doi: 10.1037/1040-3590.5.2.164

[ref83] Peer E. Brandimarte L. Samat S. Acquisti A. (2017). Beyond the Turk: alternative platforms for crowdsourcing behavioral research. J. Exp. Soc. Psychol. 70, 153–163. doi: 10.1016/j.jesp.2017.01.006

[ref84] Pennebaker J. W. Booth R. J. Boyd R. L. Francis M. E. (2015). Linguistic Inquiry and Word Count: LIWC2015. Austin, TX: Pennebaker Conglomerates.

[ref85] Poplett E. (2020). 7 creative ways to celebrate the holidays safely during COVID-19. *BCNC*. Available at: https://blog.bcbsnc.com/2020/11/7-creative-ways-to-celebrate-the-holidays-safely-during-covid-19/ (Accessed November 11, 2020).

[ref86] Ravenscraft E. (2021). It’s not just you: Everyone’s mental health is suffering. Wired. Available at: https://www.wired.com/story/mental-health-coronavirus-pandemic-tips/ (Accessed January 18, 2021).

[ref87] Reuters (2020). America celebrates scaled-back Thanksgiving as COVID-19 surges. Available at: https://www.reuters.com/article/usa-thanksgiving/america-celebrates-scaled-back-thanksgiving-as-covid-19-surges-idUSL1N2IB2I9 (Accessed November 26, 2020).

[ref88] Ryan R. M. Deci E. L. (2017). Self-Determination Theory: Basic Psychological Needs in Motivation, Development and Wellness. Guilford Press.

[ref89] Sanders L. (2021). One in nine Americans spent the 2020 holiday season alone. YouGov. Available at: https://today.yougov.com/topics/lifestyle/articles-reports/2021/01/07/americans-spent-2020-holiday-season-alone (Accessed January 7, 2021).

[ref90] Savage M. (2020). Poll reveals scale of ‘home alone’ Christmas in the UK this year. The Guardian. Available at: https://www.theguardian.com/society/2020/dec/05/poll-reveals-scale-of-home-alone-christmas-in-the-uk-this-year (Accessed December 5, 2020).

[ref91] Scholer A. A. Cornwell J. F. M. Higgins E. T. (2019a). “Regulatory focus theory and research: catching up and looking forward after 20 years,” in The Oxford Handbook of Human Motivation. 2nd *Edn*. ed. Ryan R. M. (New York, NY, USA: Oxford University Press), 1–35.

[ref92] Scholer A. A. Cornwell J. F. M. Higgins E. T. (2019b). Should we approach approach and avoid avoidance? An inquiry from different levels. Psychol. Inq. 30, 111–124. doi: 10.1080/1047840X.2019.1643667

[ref93] Scholer A. A. Ozaki Y. Higgins E. T. (2014). Inflating and deflating the self: sustaining motivational concerns through self-evaluation. J. Exp. Soc. Psychol. 51, 60–73. doi: 10.1016/j.jesp.2013.11.008

[ref94] Scholer A. A. Zou X. Fujita K. Stroessner S. J. Higgins E. T. (2010). When risk seeking becomes a motivational necessity. J. Pers. Soc. Psychol. 99, 215–231. doi: 10.1037/a0019715, PMID: 20658840

[ref95] Sheldon K. M. Hilpert J. C. (2012). The balanced measure of psychological needs (BMPN) scale: An alternative domain general measure of need satisfaction. Motiv. Emot. 36, 439–451. doi: 10.1007/s11031-012-9279-4

[ref96] Sheldon K. M. Niemiec C. P. (2006). It’s not just the amount that counts: balanced need satisfaction also affects well-being. J. Pers. Soc. Psychol. 91, 331–341. doi: 10.1037/0022-3514.91.2.331, PMID: 16881768

[ref97] Sikali K. (2020). The dangers of social distancing: how COVID-19 can reshape our social experience. J. Community Psychol. 48, 2435–2438. doi: 10.1002/jcop.22430, PMID: 32880991 PMC7461541

[ref98] Silva C. (2020). U.S. passes 12 million confirmed coronavirus cases. NPR. Available at: https://www.npr.org/sections/coronavirus-live-updates/2020/11/21/937615700/u-s-passes-12-million-confirmed-coronavirus-cases (Accessed November 21, 2020).

[ref99] Statistica Research Department (2021). Average size of a family in the US 1960–2020. Available at: https://www.statista.com/statistics/183657/average-size-of-a-family-in-the-us/ (Accessed January 20, 2020).

[ref100] Sullivan M. (2020). How to celebrate Thanksgiving during the pandemic. *The New York Times*. Available at: https://www.nytimes.com/wirecutter/blog/celebrate-thanksgiving-coronavirus/ (Accessed November 12, 2020).

[ref101] Summerville A. Roese N. J. (2008). Self-report measures of individual differences in regulatory focus: A cautionary note. J. Res. Pers. 42, 247–254. doi: 10.1016/j.jrp.2007.05.005, PMID: 18496599 PMC2390858

[ref103] Tanne J. H. (2020). Covid-19: US faces “surge upon a surge.” BMJ. Available at: https://www.bmj.com/content/371/bmj.m4693 (Accessed December 1, 2020). 10.1136/bmj.m4693 33262104

[ref104] Thanksgiving (United States) (2020). In Wikipedia. Available at: http://en.wikipedia.org/wiki/Thanksgiving_(United_States) (Accessed January 10, 2013).

[ref105] The Old Farmer’s Almanac (2020). 4 ways Canadian Thanksgiving differs from American Thanksgiving. Available at: https://www.almanac.com/content/ways-canadian-thanksgiving-differs-american-thanksgiving (Accessed October 2, 2020).

[ref106] Thomas N. (2020). 61% of Americans changed their Thanksgiving plans due to Covid-19 spikes, new poll finds. CNN. Available at: https://www.cnn.com/2020/11/24/health/thanksgiving-2020-plans-covid-wellness/index.html (Accessed November 24, 2020).

[ref107] Trotta D. Layne N. (2020). U.S. holiday travelers voice dread, determination as they defy COVID-19 warnings. Reuters. Available at: https://www.reuters.com/article/health-coronavirus-usa/update-5-many-americans-defying-covid-19-travel-warnings-ahead-of-thanksgiving-holiday-idUSL1N2I90XE (Accessed November 23, 2020).

[ref108] Vanderweele T. J. Fulks J. Plake J. F. Lee M. T. (2020). National well-being measures before and during the COVID-19 pandemic in online samples. J. Gen. Intern. Med. 36, 248–250. doi: 10.1007/s11606-020-06274-3, PMID: 33078296 PMC7571529

[ref109] Vansteenkiste M. Ryan R. M. Soenens B. (2020). Basic psychological need theory: advancements, critical themes, and future directions. Motivation Emotion. doi: 10.1007/s11031-019-09818-1

[ref110] Vaughn L. A. (2017). Foundational tests of the need-support model: A framework for bridging regulatory focus theory and self-determination theory. Personal. Soc. Psychol. Bull. 43, 313–328. doi: 10.1177/0146167216684132, PMID: 28903697

[ref111] Vaughn L. A. (2018). Contents of hopes and duties: A linguistic analysis. Front. Psychol. 9:757. doi: 10.3389/fpsyg.2018.00757, PMID: 29867701 PMC5964360

[ref112] Vaughn L. A. (2019). Distinguishing between need support and regulatory focus with LIWC. Collabra Psychol. 5:32. doi: 10.1525/collabra.185

[ref113] Vaughn L. A. Baumann J. Klemann C. (2008). Openness to experience and regulatory focus: evidence of motivation from fit. J. Res. Pers. 42, 886–894. doi: 10.1016/j.jrp.2007.11.008

[ref114] Vaughn L. Burkins P. Judd J. (2020). Exploration and self-control: Differences in regulatory focus and need support. Available at: http://psyarxiv.com/xamdu (Accessed July 28, 2020).

[ref115] Vaughn L. A. Childs K. E. Maschinski C. Niño N. P. Ellsworth R. (2010a). Regulatory fit, processing fluency, and narrative persuasion. Soc. Personal. Psychol. Compass 4, 1181–1192. doi: 10.1111/j.1751-9004.2010.00325.x

[ref116] Vaughn L. A. Garvey C. A. Chalachan R. D. (2020). Need support and regulatory focus in responding to COVID-19. Front. Psychol. 11:589446. doi: 10.3389/fpsyg.2020.589446, PMID: 33329250 PMC7717948

[ref117] Vaughn L. A. Harkness A. R. Clark E. K. (2010b). The effect of incidental experiences of regulatory fit on trust. Pers. Relat. 17, 57–69. doi: 10.1111/j.1475-6811.2010.01252.x

[ref118] Vaughn L. A. Hesse S. J. Petkova Z. Trudeau L. (2009). “This story is right on”: the impact of regulatory fit on narrative engagement and persuasion. Eur. J. Soc. Psychol. 39, 447–456. doi: 10.1002/ejsp.570

[ref119] Vaughn L. A. Malik J. Schwartz S. Petkova Z. Trudeau L. (2006a). Regulatory fit as input for stop rules. J. Pers. Soc. Psychol. 91, 601–611. doi: 10.1037/0022-3514.91.4.601, PMID: 17014287

[ref120] Vaughn L. A. O’Rourke T. Schwartz S. Malik J. Petkova Z. Trudeau L. (2006b). When two wrongs can make a right: regulatory nonfit, bias, and correction of judgments. J. Exp. Soc. Psychol. 42, 654–661. doi: 10.1016/j.jesp.2005.09.004

[ref121] Vowels L. M. Carnelley K. B. (2020). Attachment styles, negotiation of goal conflict, and perceived partner support during COVID-19. Personal. Individ. Differ. 171:110505. doi: 10.1016/j.paid.2020.110505 PMC9045812 35502309

[ref122] Wallace J. C. Johnson P. D. Frazier M. L. (2009). An examination of the factorial, construct, and predictive validity and utility of the regulatory focus at work scale. J. Organ. Behav. 30, 805–831. doi: 10.1002/job.572

[ref123] Walters K. Christakis D. A. Wright D. R. (2018). Are Mechanical Turk worker samples representative of health status and health behaviors in the U.S.? PLoS One 13:e0198835. doi: 10.1371/journal.pone.0198835, PMID: 29879207 PMC5991724

[ref124] Warm J. S. Parasuraman R. Matthews G. (2008). Vigilance requires hard mental work and is stressful. Hum. Factors 50, 433–441. doi: 10.1518/001872008X312152, PMID: 18689050

[ref125] Whitcomb D. Layne N. (2020). ‘Kind of lonely’: America marks COVID altered Thanksgiving. Reuters. Available at: https://www.reuters.com/article/usa-thanksgiving/america-celebrates-scaled-back-thanksgiving-as-covid-19-surges-idINKBN2861LH?edition-redirect=in (Accessed November 26, 2020).

[ref126] Witters D. (2011). Americans happier, less stressed in 2010. Gallup. Available at: http://www.gallup.com/poll/145457/Americans-Happier-Less-Stressed-2010.aspx (Accessed January 4, 2011).

[ref127] World Health Organization (2021). There’s a Little Hero in Us All. Fight COVID-19 with the WHO’s Five Heroic Acts. Available at: https://www.who.int/campaigns/connecting-the-world-to-combat-coronavirus/safehands-challenge/5-heroic-acts (Accessed January 19, 2021).

[ref128] Wu C. Chen W. (2020). Mediating role of regulatory focus in the relation between filial piety and youths’ life satisfaction and psychological distress. Asian J. Soc. Psychol. doi: 10.1111/ajsp.12447 [Epub ahead of print].

[ref129] Yoon Y. Sarial-Abi G. Gürhan-Canli Z. (2012). Effect of regulatory focus on selective information processing. J. Consum. Res. 39, 93–110. doi: 10.1086/661935

[ref130] Zhang Y. Zhang Y. Ng T. W. H. Lam S. S. K. (2019). Promotion- and prevention-focused coping: a meta-analytic examination of regulatory strategies in the work stress process. J. Appl. Psychol. 104, 1296–1323. doi: 10.1037/apl0000404 30945878

