# Supplementary material for: Feeling Socially Connected and Focusing on Growth: Relationships With Wellbeing During a Major Holiday in the COVID-19 Pandemic
Source: Front Psychol. 2021 Aug 26;12:710491. doi: 10.3389/fpsyg.2021.710491 (PMC8496488; doi:10.3389/fpsyg.2021.710491)
Supplement: Supplementary file 1 [file Data_Sheet_1.PDF]

Methodology Reporting for the Article:

Feeling socially connected and focusing on growth: Relationships with well-being during a major holiday in the COVID-19 pandemic

Leigh Ann Vaughn, Patricia G. Burkins, Rachael D. Chalachan, Janak K. Judd, Chase A. Garvey, John W. Luginsland

Methodology Reporting

In this document, a dashed line indicates the location of a page break in the online questionnaire.

Before you start, please switch off phone/ e-mail/ music so you can focus on this study.

Thank you!

Please enter your Prolific ID here:

How You Spent this Thanksgiving Day

First, we would like to learn about how you spent this Thanksgiving Day, 2020. This is a general question, and you can write about your thoughts, feelings, and/or behaviors.

Please take a minute or two and write about how you spent this Thanksgiving Day.

How You Spent this Thanksgiving Day

Earlier, you wrote:

[piped in what they wrote on the previous page]

Please indicate how much you agree with the following statements about how you spent this Thanksgiving Day.

(1 = strongly disagree; 4 = neither agree nor disagree; 7 = strongly agree)

- I was free to do things my own way.
- I successfully completed difficult tasks and projects.
- I felt a sense of contact with people who care for me, and whom I care for.
- I had a lot of pressures I could do without.
- I experienced some kind of failure, or was unable to do well at something.
- I was lonely.
- My choices expressed my “true self.”
- I took on and mastered hard challenges.
- I felt close and connected with other people who are important to me.
- There were people telling me what I had to do.
- I did stupid things, that made me feel incompetent.

- I felt unappreciated by one or more important people.
- I was really doing what interested me.
- I did well even at the hard things.
- I felt a strong sense of intimacy with the people I spent time with.
- I had to do things against my will.
- I struggled doing something I should be good at.
- I had disagreements or conflicts with people I usually get along with.

- I felt that my actions had a positive impact on the people around me.
- The things I did contributed to the betterment of society.
- I was able to improve the welfare of other people.
- In general, my influence in the lives of other people was positive.

How You Spent this Thanksgiving Day

Earlier, you wrote:

[piped in what they wrote on the earlier page]

Please indicate how much each of the following describes how you spent this Thanksgiving Day.

(1 = not at all, 7 = very much)

- I avoided making mistakes.
- I tried new things just because they could be interesting.
- I exerted self-control.
- I was enthusiastic.
- I thought through anything that could go wrong.
- I was optimistic.
- I fulfilled duties and obligations.
- I was spontaneous.
- I did what was expected of me.
- I avoided missing out on anything good.
- I was careful.
- I did what I ideally liked to.
- I was cautious.
- I was excited.
- I exerted willpower.
- I avoided thinking about what could go wrong.

How You Spent this Thanksgiving Day

Earlier, you wrote:

[piped in what they wrote on the earlier page]

Please think about what you did and experienced on Thanksgiving Day. Then report how much you experienced each of the following feelings. (1 = very slightly, 7 = extremely)

- Positive
- Negative
- Good
- Bad
- Pleasant
- Unpleasant
- Happy
- Sad
- Afraid
- Joyful
- Angry
- Contented

How You Spent this Thanksgiving Day

Earlier, you wrote:

[piped in what they wrote on the earlier page]

How satisfied are you with how this Thanksgiving Day went?
(1 = strongly disagree, 4 = neither agree nor disagree, 7 = strongly agree)

- In most ways this Thanksgiving Day was close to my ideal.
- The conditions of my life this Thanksgiving Day were excellent.
- I was satisfied with this Thanksgiving Day.
- I got the important things I wanted out of this Thanksgiving Day.
- If I could live this Thanksgiving Day over, I would change almost nothing.

How You Spent this Thanksgiving Day

Earlier, you wrote:

[piped in what they wrote on the earlier page]

How many other people did you see face-to-face on Thanksgiving Day this year? [slider that went from 0 to 100 in increments of 1 and showed the value]

Including you, how many people normally live in your usual residence? 1, 2, 3, 4, 5, 6, 7 or more

How many other people did you see face-to-face on Thanksgiving Day last year, in 2019? [slider that went from 0 to 100 in increments of 1 and showed the value]

Including you, how many people normally lived in your usual residence at that time last year? 1, 2, 3, 4, 5, 6, 7 or more

---

Demographic Questions

How old are you? Please write only a number, for example, 18.

What is your gender identity? (select all that apply)

- Man
- Woman
- Transgender
- Non-binary/nonconforming
- Other
- Prefer not to respond

What is your ethnic group? (you may select more than one)

Asian
Black or African-American
Hispanic or Latino
Multiethnic
Native American or Alaska Native
Native Hawaiian or Pacific Islander
White
Other

In which country were you born?

Is English your native language? Yes No

In which state do you live? [drop-down list of states]

What is the highest level of education you have completed?

What is your primary occupation?

Impressions of the Study

Thank you for participating!
What are your impressions of the study?

Were you distracted by anything going on in the room while you were doing the study? (yes/no)

If yes, participants went to a page that asked them what they were distracted by and how distracted they were.
