# Supplementary material for: Feeling Socially Connected and Focusing on Growth: Relationships With Wellbeing During a Major Holiday in the COVID-19 Pandemic
Source: Front Psychol. 2021 Aug 26;12:710491. doi: 10.3389/fpsyg.2021.710491 (PMC8496488; doi:10.3389/fpsyg.2021.710491)
Supplement: Supplementary file 2 [file Data_Sheet_2.PDF]

Online Supplement for the Article:

Feeling socially connected and focusing on growth: Relationships with well-being during a major holiday in the COVID-19 pandemic

Leigh Ann Vaughn, Patricia G. Burkins, Rachael D. Chalachan, Janak K. Judd, Chase A. Garvey, John W. Luginsland

The data files and methodology file are available at https://osf.io/fy6rc/.

This document contains the following tables and other resources.

- Page 3
    - Table S1: Multiple regressions modelling relationships from regulatory focus to need satisfaction
- Page 4
    - Table S2: Multiple regressions modelling relationships from need satisfaction to regulatory focus
- Page 5
    - Table S3: Communalities and factor loadings from the exploratory factor analysis of promotion and affect items
- Page 6
    - Table S4: Communalities and factor loadings from the exploratory factor analysis of promotion and satisfaction with Thanksgiving items
- Page 7
    - Description of the Beneficence Scale, which we included in the study for exploratory purposes. The methodology file at https://osf.io/fy6rc/ contains the entire Beneficence Scale.
- Page 8
    - Table S5: Correlations, Cronbach's alphas, means, and standard deviations, **including beneficence**
- Page 9
    - Table S6: Multiple regressions modelling relationships with positive affect, negative affect, and satisfaction with Thanksgiving, **including beneficence**
- Page 10
    - Table S7: Multiple regressions modelling relationships from regulatory focus to need satisfaction **and beneficence**
- Page 11
    - Table S8: Multiple regressions modelling relationships from need satisfaction to regulatory focus **and beneficence**
- Pages 12-13
    - Table S9: Tests of differences between participants who saw 0, 1, or 2+ other people face-to-face on Thanksgiving, **including beneficence**
- Page 14
    - References for this document

Table S1

Multiple Regressions Modelling Relationships from Regulatory Focus to Need Satisfaction

Dependent Variables and Predictors	*B*	β	sr^2	*p*	95% CI for *B*
Autonomy					
**Promotion**	**0.59**	**0.64**	**.366**	**< .001**	**[0.51, 0.67]**
Caution/self-control	-0.01	-0.01	< .001	.785	[-0.08, 0.06]
**Duties**	**-0.18**	**-0.19**	**.031**	**< .001**	**[-0.27, -0.10]**
Competence					
**Promotion**	**0.35**	**0.47**	**.199**	**< .001**	**[0.29, 0.41]**
Caution/self-control	0.06	0.08	.006	.053	[0.00, 0.12]
**Duties**	**0.16**	**0.22**	**.038**	**< .001**	**[0.10, 0.23]**
Relatedness					
**Promotion**	**0.63**	**0.66**	**.389**	**< .001**	**[0.56, 0.71]**
Caution/self-control	-0.04	-0.04	.002	.270	[-0.11, 0.03]
Duties	0.08	0.08	.006	.033	[0.01, 0.16]

Note. B = unstandardized regression weights. β = standardized regression weights. sr^2 = semi-partial correlation squared. CI = confidence interval. Rows in bold font indicate significant results with power $\geq$ .80.

Table S2

Multiple Regressions Modelling Relationships from Need Satisfaction to Regulatory Focus

Dependent Variables and Predictors	*B*	β	sr^2	*p*	95% CI for *B*
Promotion					
**Autonomy**	**0.23**	**0.21**	**.028**	**< .001**	**[0.14, 0.32]**
**Competence**	**0.26**	**0.19**	**.024**	**< .001**	**[0.15, 0.37]**
**Relatedness**	**0.47**	**0.45**	**.115**	**< .001**	**[0.38, 0.57]**
Caution/self-control					
Autonomy	-0.15	-0.13	.010	.040	[-0.29, -0.01]
**Competence**	**0.35**	**0.25**	**.040**	**< .001**	**[0.18, 0.52]**
Relatedness	-0.06	-0.06	.002	.372	[-0.20, 0.08]
Duties					
**Autonomy**	**-0.37**	**-0.35**	**.075**	**< .001**	**[-0.49, -0.25]**
**Competence**	**0.54**	**0.41**	**.112**	**< .001**	**[0.40, 0.68]**
**Relatedness**	**0.27**	**0.27**	**.040**	**< .001**	**[0.16, 0.39]**

Note. B = unstandardized regression weights. β = standardized regression weights. sr^2 = semi-partial correlation squared. CI = confidence interval. Rows in bold font indicate significant results with power $\geq$ .80.

Table S3

Communalities and Factor Loadings from the Exploratory Factor Analysis of Promotion and Positive Affect

	Factor		Communalities	
Item	1	2	Initial	Extracted
PA2: Good	**.982**	-.053	.850	.884
PA3: Pleasant	**.933**	-.045	.781	.806
PA4: Happy	**.911**	.041	.872	.891
PA1: Positive	**.877**	.068	.850	.870
PA6: Contented	**.811**	-.006	.643	.649
PA5: Joyful	**.654**	.275	.783	.788
Pro7: I was excited.	.259	**.672**	.747	.793
Pro4: I was spontaneous.	-.041	**.628**	.385	.355
Pro2: I was enthusiastic.	.311	**.610**	.740	.769
Pro1: I tried new things just because they could be interesting.	-.062	**.557**	.315	.259
Pro5: I avoided missing out on anything good.	.080	**.478**	.303	.296
Pro3: I was optimistic.	**.439**	**.464**	.720	.732
Pro6: I did what I ideally liked to.	.370	.397	.567	.527

Note: *N* = 404. Pro = promotion. PA = positive affect. Maximum likelihood exploratory factor analysis and direct oblimin rotation with delta = 0. Loadings are from the pattern matrix, and loadings over .40 are in bold font. Factor 1 represents positive affect, and Factor 2 represents promotion focus.

Table S4

Communalities and Factor Loadings from the Exploratory Factor Analysis of Promotion and Satisfaction with Thanksgiving Items

Item	Factor		Communalities	
	1	2	Initial	Extracted
Pro2: I was enthusiastic.	**.911**	.018	.738	.804
Pro3: I was optimistic.	**.900**	.051	.678	.738
Pro7: I was excited.	**.786**	-.109	.731	.769
Pro4: I was spontaneous.	**.550**	-.020	.382	.321
Pro1: I tried new things just because they could be interesting.	**.504**	.025	.313	.234
Pro5: I avoided missing out on anything good.	**.430**	-.129	.304	.291
Pro6: I did what I ideally liked to.	.257	**-.545**	.624	.589
SWTS1: In most ways this Thanksgiving Day was close to my ideal.	-.078	**-.921**	.690	.738
SWTS2: The conditions of my life this Thanksgiving Day were excellent.	-.020	**-.843**	.659	.684
SWTS4: I got the important things I wanted out of this Thanksgiving Day.	.064	**-.808**	.719	.740
SWTS3: I was satisfied with this Thanksgiving Day.	.171	**-.769**	.800	.834
SWTS5: If I could live this Thanksgiving Day over, I would change almost nothing.	-.029	**-.764**	.526	.549

Note: *N* = 404. Pro = promotion. SWTS = Satisfaction with Thanksgiving Scale. Maximum likelihood exploratory factor analysis and direct oblimin rotation with delta = 0. Loadings are from the pattern matrix, and loadings over .40 are in bold font. Factor 1 represents promotion focus, and Factor 2 represents satisfaction with Thanksgiving.

Information about the Beneficence Scale (Martela & Ryan, 2016)

For exploratory purposes, we assessed satisfaction of the candidate need for beneficence – that is, for making a positive difference in others' lives (Martela & Ryan, 2016b). Research continues to assess whether beneficence is truly a need or is more a well-being enhancer (Martela & Ryan, 2016a, 2016b, 2020; Martela et al., 2018). We included it for two reasons. One is that helping others is an important Thanksgiving tradition ('Thanksgiving (United States),' n.d.). The other is that research on responding to COVID-19 in the first month of the pandemic suggested that people responded to the virus with the most enthusiasm when they felt that they could help others (Vaughn, Garvey, & Chalachan, 2020).

On the second page of stimulus materials, the last four statements were the Beneficence Scale (Martela & Ryan, 2016b). It includes statements such as, "I felt that my actions had a positive impact on the people around me," and "The things I did contributed to the betterment of society." Tables S5-S9 show the results of analyses including the Beneficence Scale.

The Beneficence Scale did not relate to well-being when controlling for relationships with measures of autonomy, competence, and relatedness satisfaction and regulatory focus. Additionally, participants who saw no one else face-to-face on Thanksgiving reported significantly lower beneficence than participants who saw either one other person or two or more other people face-to-face that day. The latter two groups did not differ significantly.

We speculate that because of the pandemic, people could not participate in the usual holiday traditions of volunteering and doing other things to help strangers. This may have been why beneficence did not relate substantially to need satisfaction or well-being on Thanksgiving in 2020 when controlling for relationships with the other predictors. Additionally, being alone on Thanksgiving may have reflected or caused low beneficence on that day.

Table S5

Correlations, Cronbach's alphas, means, and standard deviations

Variable	1	*2*	*3*	4	5	6	7	8	9	10
1. Promotion	-									
2. Caution	.03	-								
3. Duties	.32**	.27**	-							
4. Autonomy	.58**	-.04	.01	-						
5. Competence	.54**	.15**	.39**	.49**	-					
6. Relatedness	.69**	.00	.29**	.60**	.55**	-				
7. Beneficence	.56**	.19**	.45**	.34**	.47**	.51**	-			
8. Pos. affect	.82**	.01	.30**	.60**	.52**	.78**	.50**	-		
9. Neg. affect	-.59**	.10*	-.15**	-.58**	-.48**	-.68**	-.37**	-.77**	-	
10. SWTS	.77**	-.07	.24**	.56**	.44**	.70**	.43**	.80**	-.65**	-
Cronbach's α	.87	.72	.69	.81	.65	.82	.86	.96	.92	.92
M	4.37	5.07	5.22	5.18	4.98	5.42	4.61	5.21	2.29	4.59
SD	(1.32)	(1.38)	(1.28)	(1.21)	(0.97)	(1.26)	(1.29)	(1.47)	(1.29)	(1.66)

Note. * indicates $p < .05$. ** indicates $p < .01$. SWTS = Satisfaction with Thanksgiving Scale. *M* and *SD* represent mean and standard deviation, respectively.

Table S6

Multiple Regressions Modelling Relationships with Positive Affect, Negative Affect, and Satisfaction with Thanksgiving

Dependent Variables and Predictors	*B*	β	sr^2	*p*	95% CI for *B*
Positive Affect					
**Promotion**	**0.57**	**0.51**	**.11**	**< .001**	**[0.49, 0.66]**
Caution/self-control	< 0.01	< 0.01	< .01	.891	[-0.06, 0.05]
Duties	0.06	0.05	< .01	.081	[-0.01, 0.13]
Autonomy	0.12	0.10	.01	.003	[0.04, 0.20]
Competence	-0.04	-0.02	< .01	.471	[-0.13, 0.06]
**Relatedness**	**0.44**	**0.38**	**.06**	**< .001**	**[0.35, 0.52]**
Beneficence	-0.03	-0.03	< .01	.353	[-0.11, 0.04]
Negative Affect					
Promotion	-0.17	-0.17	.01	.002	[-0.27, -0.06]
Caution/self-control	0.10	0.11	.01	.003	[0.03, 0.17]
Duties	0.02	0.02	< .01	.598	[-0.06, 0.11]
**Autonomy**	**-0.21**	**-0.19**	**.02**	**< .001**	**[-0.31, -0.11]**
Competence	-0.14	-0.10	.01	.028	[-0.26, -0.01]
**Relatedness**	**-0.41**	**-0.40**	**.07**	**< .001**	**[-0.52, -0.31]**
Beneficence	0.02	0.02	< .01	.735	[-0.07, 0.10]
Satisfaction with Thanksgiving					
**Promotion**	**0.69**	**0.54**	**.12**	**< .001**	**[0.57, 0.80]**
Caution/self-control	-0.08	-0.07	< .01	.026	[-0.16, -0.01]
Duties	0.04	0.03	< .01	.350	[-0.05, 0.14]
Autonomy	0.14	0.11	.01	.011	[0.03, 0.25]
Competence	-0.09	-0.05	< .01	.190	[-0.22, 0.04]
**Relatedness**	**0.40**	**0.31**	**.04**	**< .001**	**[0.29, 0.52]**
Beneficence	-0.06	-0.05	< .01	.227	[-0.16, 0.04]

Note. B = unstandardized regression weights. β = standardized regression weights. sr^2 = semi-partial correlation squared. CI = confidence interval. Rows in bold font indicate significant results with power $\geq$ .80.

Table S6

Multiple Regressions Modelling Relationships from Regulatory Focus to Need Satisfaction

Dependent Variables and Predictors	*B*	β	sr^2	*p*	95% CI for *B*
Autonomy satisfaction					
**Promotion**	**0.59**	**0.64**	**.37**	**< .001**	**[0.51, 0.67]**
Caution/self-control	-0.01	-0.01	< .01	.785	[-0.08, 0.06]
**Duties**	**-0.18**	**-0.19**	**.03**	**< .001**	**[-0.27, -0.10]**
Competence satisfaction					
**Promotion**	**0.35**	**0.47**	**.20**	**< .001**	**[0.29, 0.41]**
Caution/self-control	0.06	0.08	.01	.053	[0.00, 0.12]
**Duties**	**0.16**	**0.22**	**.04**	**< .001**	**[0.10, 0.23]**
Relatedness satisfaction					
**Promotion**	**0.63**	**0.66**	**.41**	**< .001**	**[0.56, 0.71]**
Caution/self-control	-0.04	-0.04	< .01	.270	[-0.11, 0.03]
Duties	0.08	0.08	.08	.033	[0.01, 0.16]
Beneficence satisfaction					
**Promotion**	**0.46**	**0.47**	**.20**	**< .001**	**[0.38, 0.54]**
Caution/self-control	0.10	0.11	.01	.007	[0.03, 0.18]
**Duties**	**0.27**	**0.27**	**.06**	**< .001**	**[0.19, 0.35]**

Note. *B* = unstandardized regression weights. β = standardized regression weights. sr^2 = semi-partial correlation squared. CI = confidence interval. Rows in bold font indicate significant results with power $\geq$ .80.

Table S7

Multiple Regressions Modelling Relationships from Need Satisfaction to Regulatory Focus

Dependent Variables and Predictors	*B*	β	sr^2	*p*	95% CI for *B*
Promotion					
**Autonomy**	**0.24**	**0.22**	**.03**	**< .001**	**[0.15, 0.32]**
Competence	0.17	0.12	.01	.003	[0.06, 0.28]
**Relatedness**	**0.38**	**0.37**	**.07**	**< .001**	**[0.29, 0.47]**
**Beneficence**	**0.25**	**0.24**	**.04**	**< .001**	**[0.17, 0.33]**
Caution/self-control					
Autonomy	-0.14	-0.13	.01	.042	[-0.28, -0.01]
**Competence**	**0.27**	**0.19**	**.02**	**.002**	**[0.09, 0.44]**
Relatedness	-0.15	-0.14	.01	.041	[-0.30, -0.01]
**Beneficence**	**0.23**	**0.22**	**.03**	**< .001**	**[0.11, 0.35]**
Duties					
**Autonomy**	**-0.36**	**-0.35**	**.07**	**< .001**	**[-0.47, -0.25]**
**Competence**	**0.42**	**0.32**	**.06**	**< .001**	**[0.28, 0.56]**
Relatedness	0.15	0.14	.01	.015	[0.03, 0.26]
**Beneficence**	**0.34**	**0.34**	**.08**	**< .001**	**[0.24, 0.44]**

Note. B = unstandardized regression weights. β = standardized regression weights. sr^2 = semi-partial correlation squared. CI = confidence interval. Rows in bold font indicate significant results with power $\geq$ .80.

Table S8

Tests of Differences between Participants Who Saw 0, 1, or 2+ Other People Face-to-Face on Thanksgiving

Measure and test	*df*s	*F*	*P*	η^2	Mean diff.	Sig.	95% CI
**Autonomy**	**(2, 401)**	**9.37**	**< .001**	**.045**			
0 vs. 1 other person					0.38	.482	[-0.27, 1.04]
0 vs. 2+ others					-0.31	.570	[-0.89, 0.26]
**1 vs. 2+ others**					**-0.70**	**< .001**	**[-1.09, -0.30]**
Competence	(2, 401)	.31	.733	.002			
0 vs. 1 other person					0.17	1.000	[-0.36, 0.71]
0 vs. 2+ others					0.13	1.000	[-0.33, 0.60]
1 vs. 2+ others					-0.04	1.000	[-0.36, 0.28]
**Relatedness**	**(2, 401)**	**11.74**	**< .001**	**.055**			
**0 vs. 1 other person**					**1.21**	**< .001**	**[0.53, 1.89]**
**0 vs. 2+ others**					**1.18**	**< .001**	**[0.59, 1.78]**
1 vs. 2+ others					-0.03	1.000	[-0.44, 0.38]
**Beneficence**	**(2, 401)**	**5.29**	**.005**	**.026**			
**0 vs. 1 other person**					**0.88**	**.009**	**[0.17, 1.59]**
**0 vs. 2+ others**					**0.81**	**.005**	**[0.20, 1.43]**
1 vs. 2+ others					-0.07	1.000	[-0.49, 0.36]
**Promotion**	**(2, 401)**	**7.90**	**< .001**	**.038**			
**0 vs. 1 other person**					**1.05**	**.001**	**[0.33, 1.76]**
**0 vs. 2+ others**					**1.02**	**< .001**	**[0.40, 1.64]**
1 vs. 2+ others					-0.03	1.000	[-0.46, 0.40]
**Caution/self-control**	**(2, 401)**	**3.25**	**.040**	**.016**			
0 vs. 1 other person					-0.26	1.000	[-1.02, 0.50]
0 vs. 2+ others					-0.58	.108	[-1.24, 0.08]
1 vs. 2+ others					-0.32	.286	[-0.77, 0.14]
**Duties**	**(2, 401)**	**22.30**	**< .001**	**.100**			
0 vs. 1 other person					0.62	.079	[-0.05, 1.30]
**0 vs. 2+ others**					**1.35**	**< .001**	**[0.77, 1.94]**
**1 vs. 2+ others**					**0.73**	**< .001**	**[0.33, 1.13]**
**Positive Affect**	**(2, 401)**	**6.03**	**.003**	**.029**			
**0 vs. 1 other person**					**0.96**	**.013**	**[0.16, 1.76]**
**0 vs. 2+ others**					**1.01**	**.002**	**[0.31, 1.71]**
1 vs. 2+ others					0.05	1.000	[-0.43, 0.53]
Negative Affect	(2, 401)	2.30	.102	.011			
0 vs. 1 other person					-0.57	.167	[-1.28, 0.14]
0 vs. 2+ others					-0.54	.106	[-1.16, 0.08]
1 vs. 2+ others					0.02	1.000	[-0.40, 0.45]
**Satisfaction with Thanksgiving**	**(2, 401)**	**10.80**	**< .001**	**.051**			
**0 vs. 1 other person**					**1.51**	**< .001**	**[0.62, 2.41]**
**0 vs. 2+ others**					**1.50**	**< .001**	**[0.72, 2.28]**

1 vs. 2+ others	-0.01	1.000	[-0.55, 0.52]

Note: 2+ others = saw two or more other people face-to-face. Group sizes: saw 0 other people (*n* = 27), 1 other person (*n* = 63), 2+ others (*n* = 314). Bonferroni post-hoc tests, in which numbers indicate higher means for the second condition within the pair. Mean diff. = mean difference between conditions. CI = confidence interval. Bold font indicates rows with significant effects.

References

Martela, F., & Ryan, R. M. (2016a). Prosocial behavior increases well-being and vitality even without contact with the beneficiary: Causal and behavioral evidence. *Motivation and Emotion*, *40*, 351–357. https://doi.org/10.1007/s11031-016-9552-z

Martela, F., & Ryan, R. M. (2016b). The benefits of benevolence: Basic psychological needs. beneficence, and the enhancement of well-being. *Journal of Personality*, *84*(6), 750–764. https://doi.org/10.1111/jopy.12215

Martela, F., & Ryan, R. M. (2020). Distinguishing between basic psychological needs and basic wellness enhancers: The case of beneficence as a candidate psychological need. *Motivation and Emotion*, *44*, 116–133. https://doi.org/10.1007/s11031-019-09800-x

Martela, F., Ryan, R. M., & Steger, M. F. (2018). Meaningfulness as satisfaction of autonomy, competence, relatedness, and beneficence: Comparing the four satisfactions and positive affect as predictors of meaning in life. *Journal of Happiness Studies*, *19*, 1261–1282. https://doi.org/10.1007/s1090 2-017-9869-7

Thanksgiving (United States). (n.d.). In Wikipedia. Retrieved January 10, 2013 from http://en.wikipedia.org/wiki/Thanksgiving_(United_States)

Vaughn, L. A., Garvey, C. A., & Chalachan, R. D. (2020). Need support and regulatory focus in responding to COVID-19. *Frontiers in Psychology*, *11*(589446), 1-13. https://doi.org/10.3389/fpsyg.2020.589446
